# Diet–Gut Microbiota Relations: Critical Appraisal of Evidence From Studies Using Metagenomics

**DOI:** 10.1093/nutrit/nuae192

**Published:** 2024-12-24

**Authors:** Mrunalini Lotankar, Noora Houttu, Kati Mokkala, Kirsi Laitinen

**Affiliations:** Integrative Physiology and Pharmacology Unit, Institute of Biomedicine, Faculty of Medicine, University of Turku, 20520 Turku, Finland; Integrative Physiology and Pharmacology Unit, Institute of Biomedicine, Faculty of Medicine, University of Turku, 20520 Turku, Finland; Integrative Physiology and Pharmacology Unit, Institute of Biomedicine, Faculty of Medicine, University of Turku, 20520 Turku, Finland; Nutrition and Food Research Center, Faculty of Medicine, University of Turku, 20520 Turku, Finland; Integrative Physiology and Pharmacology Unit, Institute of Biomedicine, Faculty of Medicine, University of Turku, 20520 Turku, Finland; Nutrition and Food Research Center, Faculty of Medicine, University of Turku, 20520 Turku, Finland; Department of Obstetrics and Gynecology, Turku University Hospital, Wellbeing Services County of Southwest Finland, 20520 Turku, Finland

**Keywords:** diet, dietary pattern, gut microbiota, metagenomics, shotgun sequencing, intervention

## Abstract

Diet may influence the gut microbiota and subsequently affect the host’s health. Recent developments in methods analyzing the composition and function of the gut microbiota allow a deeper understanding of diet–gut microbiota relationships. A state-of-the-art methodology, shotgun metagenomics sequencing, offers a higher taxonomic resolution of the gut microbiota at the bacterial species and strain levels, and more accurate information regarding the functional potential of gut microbiota. Here, the available evidence on the relationship between diet and gut microbiota was critically reviewed, focusing on results emerging from recent metagenomics sequencing studies applied in randomized controlled trials and observational studies. The PubMed and Embase databases were used to search publications between January 2011 and September 2023. Thus far, the number of studies is limited, and the study designs and methods utilized have been variable. Nevertheless, the cumulative evidence from interventions relates to dietary fiber as a modifier of bacterial species, such as *Anaerostipes hadrus* and *Faecalibacterium prausnitzii*. Furthermore, observational studies have detected associations between different dietary patterns and food groups with certain microbial species. Utilization of metagenomics sequencing is becoming more common and will undoubtedly provide further insights into diet–gut microbiota relationships at the species level as well as their functional pathways in the near future. For reproducible results and to draw reliable conclusions across various studies on diet–gut microbiota relationships, there is a need for harmonization of the study designs and standardized ways of reporting.

## INTRODUCTION

The gut microbiota, a diverse and complex community of microorganisms, plays a significant role in overall human health. It contributes to the nutrient and energy metabolism not only via the synthesis of vitamins and amino acids but also via the production of short-chain fatty acids (SCFAs), such as butyrate, acetate, and propionate.^1–3^ Although not clearly defined, in general, a gut microbiota that is considered healthy is represented predominantly by the presence of the following phyla: Bacillota (formerly, Firmicutes),^4^ Bacteroidota (Bacteroides), and Actinomycetota (Actinobacteria), with lower abundances of Verrucomicrobiota (Verrucomicrobia) and Pseudomonadota (Proteobacteria).^5^^,^^6^ The term “dysbiosis” refers to a perturbance of the composition of the gut microbiota; most often this is evident as reduced overall microbial species diversity (eg, a decrease in butyrate-producing bacteria but an increase in the numbers of opportunistic pathogenic bacteria).^6^ Gut dysbiosis has been linked to an increased risk of cardiovascular diseases and metabolic disorders such as obesity or type 2 diabetes.^2^^,^^7^^,^^8^

A range of factors may influence the composition and function of the gut microbiota. These include age, geography, diet, stress, physical activity, intake of antibiotics, alcohol consumption, and smoking.^9^^,^^10^ Here the focus is on diet and indeed previous reports have demonstrated that variations in diet and duration of dietary exposure can affect the overall abundances of various bacteria as well as the functionality of gut microbiota.^11–14^ Most of these studies used 16S rRNA sequencing as the primary method to analyze the gut microbiota as it is the most routinely used technique due to its cost-effectiveness, availability of large reference databases, and established pipelines for data analysis.^15^ However, this method does have a few disadvantages: (1) a robust identification of bacteria but only at the taxonomical levels of phyla and genus and (2) amplification of a specific bacterial region often means that the genes regulating function cannot be identified.^15^ These limitations can be overcome by shotgun metagenomics. Metagenomics amplifies all of the genomes present in a sample and consequently provides more detailed information, for example, on bacterial species/strain levels and functional potential of the gut microbiota, topics currently of interest to many researchers.

The aim of this review was to understand the status of and knowledge on diet–gut microbiota relationships by summarizing the evidence emerging from the studies that applied metagenomics in the analysis of the gut microbiota. This review also suggests that there is a need for harmonization of the study designs and standardized ways of reporting, which can allow reproducible results and help draw reliable conclusions on diet–gut microbiota relations. A better understanding of the diet–gut microbiota relations would be advantageous when designing novel dietary approaches to modify the gut microbiota and to study the related health benefits.

## METHODS

**Figure 1.**
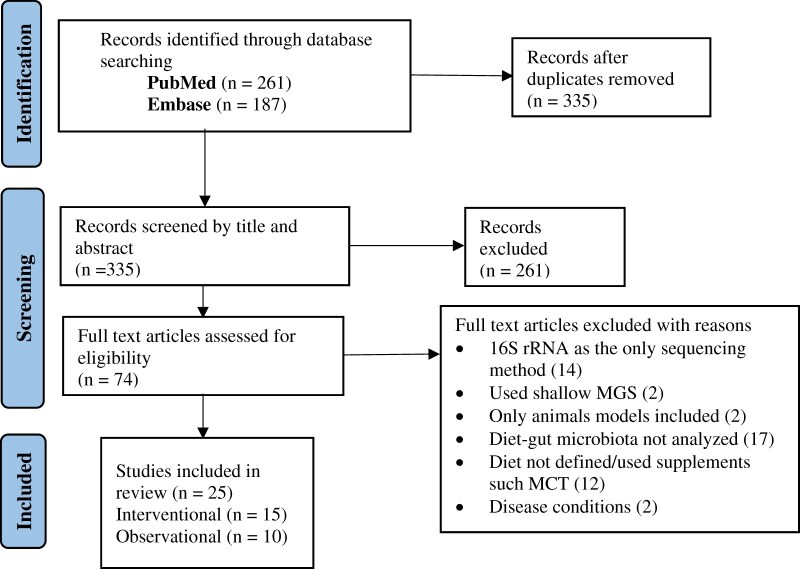
PRISMA Flowchart for the Selection of the Articles for the Current Review. Abbreviations: MCT, medium-chain triglycerides; MGS, metagenomics sequencing; PRISMA, Preferred Reporting Items for Systematic Reviews and Meta-Analyses

A search was conducted for articles using the terms “diet* AND microbio* AND metagenom* NOT review” in PubMed and Embase. The inclusion criteria for research papers were as follows: (1) primary focus on the diet–gut microbiota relations in adult humans and (2) gut microbiota analyses based on metagenomics. Studies that applied metagenomics to confirm 16S rRNA findings were also included. Studies that used 16S rRNA as the only sequencing method for gut microbiota analysis, those that focused on disease conditions, or used only animal models were excluded. A total of 33 articles were included for this review from the years January 2011 to September 2023; 16 were intervention trials and 17 were observational studies (Figure 1).

## RESULTS

The current review presents the results of the diet–gut microbiota associations based on study design—that is, either intervention (Table 1)^16–31^ or observational (Table 2)^32–48^ studies. In each section, the following topics will be briefly described: the study design, diet (intervention, diet components, or dietary pattern; observational studies), and their association with the gut microbiota with regard to alpha (α)-diversity (ie, within community diversity), beta (β)-diversity (ie, between-communities diversity), abundances of bacterial taxa, specifically bacterial species, and their predicted functions (metabolic pathways).

**Table 1.** Details of the Intervention Studies Included in the Review

Study, year, country	Primary objectives	Population (*n*); study design; duration	Diet as an intervention	Diet and microbial diversity	Diet and microbial composition
Ang et al,^16^ 2020, USA	To show that ketogenic diets alter the human and mouse gut microbiota in a manner distinct from high-fat diets	Adult overweight or class I obese, nondiabetic men (17); age: 18-50 y; BMI: 25-35 kg/m^2^; in-patient crossover study; 8 wk	BD (4 wk): 50% CHO, 15% protein, 35% fat; followed KD (4 wk): 5% CHO, 15% protein, 80% fat	—	KD: ↓Bifidobacteria spp
Asnicar et al,^17^ 2021, Italy	To identify both individual components of the microbiome and an overall gut microbial signature associated with multiple measures of dietary intake and cardiometabolic health	Generally healthy adults (UK; non-twins, as well as identical and nonidentical twins) (1002) and healthy adults from the United States (non-twins; validation cohort) (100); age: 18-65 y; single-arm, single-blinded intervention study; June 2018–May 2019	Baseline (day 1): Fasting before visit and standardized metabolic challenge meal for breakfast (0 h; 86 g CHO, 53 g fat) and lunch (4 h; 71g CHO, 22 g fat) Home phase (days 2–14): Standardized test meals in duplicate varying in sequence and in macronutrient composition	α-Diversity: Habitual diet^a^	↑ Full-fat yogurt consumption ↑ *Bifidobacterium animalis* and *Streptococcus thermophilus* Coffee consumption: *Lawsonibacter asaccharolyticus*^a^ Healthy plant-based foods: *Roseburia hominis, Agathobaculum butyriciproducens, Faecalibacterium prausnitzii*, *Anaerostipes hadrus*, *Roseburia* CAG182, and *Firmicutes* CAG95^a^ Less-healthy plant-based and animal-based foods: *Clostridium innocuum*, *C symbiosum*, *Thomasclavelia spiroformis* (formerly, *Clostridium spiroforme*), *C leptum, Lacrimispora saccharolytica* (formerly*, Clostridium saccharolyticum*)^a^ Poor dietary patterns: *Clostridium* CAG58*, Flavonifractor plautii*^a^ Intake of healthier foods and healthy patterns: *F prausnitzii*^a^
Barber et al,^18^ 2021, Spain	To determine the effect of diet on gut microbiota, digestive function and sensations, using an integrated approach involving clinical data, metagenomics, and metabolomics	Healthy men (20); age: 18-38 y; BMI: 19.2-25.5 kg/m^2^; single-center, crossover, randomized, open-label study; 8 wk	High-residue FMD: 19% fat, 62% CHO, and 16% protein with 54.2 g fiber High-fat WD: 51% fat, 27% CHO, and 21% proteins with 4.7 g fiber Washout diet: 23% fat, 55% CHO, 22% protein; 2 wk before high-residue and high-fat diets	No change	After FMD: ↑ *Agathobaculum* and *Anaerostipes* genus; species: *Agathobaculum butyriciproducens*, *Anaerostipes hadrus*
Basolo et al,^19^ 2020, USA	To study the effects of a dietary and pharmacological intervention on stool calorie loss; to evaluate the changes in the gut microbiota community structure as evidenced by amplicon sequencing and metagenomics	Healthy (27: M =17, F = 10), except that they could have impaired glucose tolerance and obesity; age: 35.1±7.3 y; BMI: 32.3±8.0 kg/m^2^; randomized crossover	WMD: 20% kcal protein, 30% kcal fat, 50% kcal CHO, adjusted to maintain a stable weight (±1%) OF diet (3-d): 150% WMD UF diet (3-d): 50% of WMD 3-d washout period in between	Relatively stable microbial diversity: 16 d ↑ Diversity: On the final day of UF with respect to OF (final day)	During UF: ↑ *Akkermansia muciniphila*, 4 *Alistipes* species
Benítez-Páez et al,^20^ 2021, Spain	To present a complementary analysis based on a multivariate multi-omics approach of a caloric-restriction intervention with fiber supplementation to unveil synergic effects on body weight control, lipid metabolism, and gut microbiota	Overweight or obese healthy male and female (80); age: 18-60 y; BMI: 28-45 kg/m^2^; randomized, double-blinded, 2-arm parallel intervention trial; June 2017–May 2018 (12 wk)	Control: Placebo supplement (CAPSA FOOD, Granda-Asturias, Spain) with same caloric content, taste, and appearance as fiber supplement (CAPSA FOOD, Granda-Asturias, Spain) Intervention: CRD + fiber supplement with 10 g/d inulin (Fibruline Instant, from chicory and average polymerization degree ∼10) +10 g/d resistant-maltodextrin (Fibersol - 2, from corn); CRD plus placebo (maltodextrin, isocaloric content) supplement: addition of fiber and placebo supplement to 200 mL of semi-skimmed milk and given twice per day (morning and afternoon)	No changes in diversity at week 12: CRD + supplement β-Diversity: Influenced by the supplement, but not the CRD intervention	CRD: ↓ *Bacteroides* species (*B cellulosilyticus*, *B timonensis*, and *B fragilis*), *Ruminococcus torques* and *Roseburia intestinalis* CRD: ↑ *Bifidobacterium longum* Fiber supplementation: ↑ *Bifidobacterium adolescentis* and *Parabacteroides distasonis* CRD + fiber supplementation: ↑ *P distasonis* (equally in men and women) ↑ *B adolescentis* and *B longum* (more in men than in women) ↓ *R torques*, *R intestinalis*, and *Coprococcus comes* (more in women than in men)
Cotillard et al,^21^ 2013, France	To investigate the temporal relationships between food intake, gut microbiota, and metabolic and inflammatory phenotypes	Overweight and obese men and women (49: M = 8, F = 41; overweight = 11, obese = 38); BMI: 33.21±0.55 kg/m^2^; follow-up study, controlled dietary intervention	Energy-restricted high-protein diet (6 wk): 1200 kcal/d for F, 1500 kcal/d for M, 35% protein, 25% lipids, 44% CHO with low glycemic index CHO and enrichment with soluble fibers Followed by WMD (6 wk): Body weight stabilization period with 20% increase in total energy intake, above resting energy metabolic rate of participants	↑ Gene richness: Fruit and vegetable intake ↑ Gene richness increased in LGC after energy-restricted diet and remained higher after stabilization phase as compared with baseline	—
Hansen et al,^22^ 2018, Denmark	To understand if a low-gluten diet affects the taxonomic and functional microbiome and host physiology in healthy individuals	Nondiabetic, lean, overweight or obese healthy White Danish adults (M = 23, F = 31); age: 22-65 y; BMI: 25-35 kg/m^2^; randomized, controlled crossover; approximately 22 wk	Two dietary intervention periods: low-gluten intake (8 wk; 2 g/d); high-gluten intake (8 wk; 18 g/d) separated by a washout period of at least 6 wk with habitual diet Habitual gluten intake: 12 g/d	α- and β-diversity: No changes	Low-gluten diet (vs high gluten diet): ↓ 4 species of *Bifidobacterium*, *Dorea longicatena*, *Blautia wexlerae*, Lachnospiraceae family, *Anaerostipes hadrus*, and *Eubacterium hallii* ↑ Unclassified species of Clostridiales and Lachnospiraceae
Li et al,^23^ 2017, China	To profile the gut microbiome in response to changes in the staple food	Mongolian participants (26); age: 25-35 y; BMI: 16-29 kg/m^2^; single arm study; 3 wk	Designed recipes Staple CHO changes—week 1: wheat; week 2: rice; week 3: oats	Weighted UniFrac distance between groups at different time points: ↑ impact of wheat, followed by rice and oats	Week 1 (wheat consumption): ↑ *Bifidobacterium catenulatum*, *Bifidobacterium bifidum*, and *Alistipes indistinctus* ↓ *Lactobacillus delbrueckii*, *Ruminococcus gnavus*, *Bacteroides vulgatus*, and *Bacteroides massiliensis* Week 2 (rice consumption): ↓ *Bifidobacterium adolescentis*, *Bifidobacterium longum*, *Weissella cibaria*, and *Rothia mucilaginosa* Week 3 (oat consumption): ↑ *Bifidobacterium adolescentis*
Meslier et al,^24^ 2020, France	To explore the effects of an MD intervention on metabolic health, gut microbiome, and systemic metabolome in individuals with lifestyle risk factors for metabolic disease	Healthy obese and overweight men and women (M = 39, F = 43), age: 20-65 y; BMI ≥24 kg/m^2^; parallel, randomized, controlled trial; 8 wk	MedD (individually tailored diet): Fruit and vegetables and nuts (at least 5 portions, ∼500 g/d), nuts (30 g/d), whole-grain products (at least 2 portions, ∼200 g/d between whole-grain pasta, bread, and breakfast cereal); fish and legumes (at least 2 portions, ∼300 g/wk of fish, and 3 portions, ∼300 g/wk of legumes); extra-virgin olive oil ConD (habitual diet): Refined cereal products; meat, eggs, and dairy products; butter/margarine	—	Intervention (vs control): ↓ *Ruthenibacterium lactatiformans*, *Flavonifractor plautii*, *Parabacteroides merdae*, *Ruminococcus torques* and *Ruminococcus gnavus*, *Streptococcus thermophilu*s ↑ *Faecalibacterium prausnitzii* clade spp, *Roseburia* and *Lachnospiraceae* spp
Oliver et al,^25^ 2021, USA	To answer 3 questions: (1) does a diet rich in fiber from whole foods alter the overall microbiome, (2) does the intervention alter the abundance and diversity of known fiber degraders, and (3) if compositional shifts are observed in the microbiome, do these correspond with metabolic changes in the production of short-chain fatty acids?	UC (University of California) Irvine students and instructors, healthy young adults (20); single arm study; 3 wk	High-fiber diet intervention: 10 meals/wk, with 15 g of fiber, 5.8 unique fruits or vegetables per meal	↓ α-Diversity (Shannon diversity): High-fiber diet intervention Changes in β-diversity: High-fiber diet^a^	↑ Fiber intake: (–) family *Lachnospiraceae*; (+) *Coprococcus* sp and *Anaerostipes hadrus* High-fiber diet: ↑ *Bifidobacterium adolescentis*, *B biavatii*, *B breve*, *B longum*, and *B ruminantium*
Rehner et al,^26^ 2023, Germany	To analyze the effects of following the PH diet over the course of 12 wk on overall biodiversity and gut microbiota composition in contrast to the most prevalent OV diet and the VV diet	41 healthy volunteers; parallel study; 12 wk	Intervention group: PH diet (details online, https://www.wwf.de/fileadmin/fm-wwf/Publikationen-PDF/Landwirtschaft/wwf-wochenmenue-besseresser-innen-flexitarisch.pdf) Control diet: Vegan/vegetarian diet and omnivorous diet (details not specified)	α- and β-diversity: No changes	VV at 0 wk: ↑ *Bifidobacterium* sp (8% *Bifidobacterium adolescentis*), *Prevotella* spp, and *Gemmiger* spp PH diet: ↑ *Prevotella copri*, *Paraprevotella xylaniphila*, *Bacteroides clarus* ↓ Firmicutes
Rinott et al,^27^ 2021, Israel	To evaluate the efficacy and safety of diet-modulated autologous fecal microbiota transplantation (aFMT) for treatment of weight regain after the weight-loss phase	Abdominally obese M and F (90); age: >30 y; waist circumference >102 cm in M and 88 cm in F; Or dyslipidemia (TG 150 and HDL-C ≤40 for M; ≤50 for F); randomized, open-label, lifestyle intervention; 6 mo	Isocaloric Mediterranean: 28 g/d walnuts (+440 mg/d polyphenols provided) Green-Mediterranean diet: Green tea (3-4 cups/d) and a Wolffia-globosa (Mankai strain; 100 g/d) green shake (+800 mg/d polyphenols provided)	Significant shift in the microbial composition observed between 0- and 6-mo fecal samples only in the green Mediterranean group	During the weight-loss phase: ↑ *Akkermansia muciniphila* Mediterranean group: ↓ *Ligilactobacillus ruminis* (formerly, *Lactobacillus ruminis*) Green Mediterranean group: ↑ *Bacteroides massiliensis*, *Paraprevotella clara*, *Alistipes putredinis*, and *Bacteroides vulgatus*
Roager et al,^28^ 2019, Denmark	To investigate whether a whole-grain diet alters the gut microbiome and insulin sensitivity, as well as biomarkers of metabolic health and gut functionality	M = 18, F = 32; age: 20-65 y; BMI: 25-35 kg/m^2^; randomized, controlled crossover; approximately 22 wk	Whole-grain diet (8 wk): 179 ± 50 g/d, separated by a washout period of at least 6 wk Refined-grain diet (8 wk): 13 ± 10 g/d	No changes in richness and diversity (refined-grain vs whole-grain)	After whole-grain: ↑ Strains of *Faecalibacterium prausnitzii* and *Prevotella copri* ↓ *Bacteroides thetaiotaomicron* After refined grain consumption: ↓ Strains of *F prausnitzii* and *P.copri* ↑ *B thetaiotaomicron*
Wu et al,^29^ 2011, USA	To investigate the association of dietary and environmental variables with the gut microbiota	Healthy men and women (M = 43, F = 55); age: 18-40 y; BMI: 18.5-35 kg/m^2^; controlled, randomized and parallel; 10 d	CAFE: Low-fiber/high-fat diet: Total calories: 38% fat, 35% CHO, and 27% protein High-fiber/low-fat diet: Total calories: 13% fat, 69% CHO, and 18% protein	No changes: Intervention (vs control)	**—**
Zhang et al,^30^ 2018, Sweden	To investigate the effect of a 3-mo lacto-ovo-vegetarian diet on the diversity of gut microbiota and the immune system in healthy omnivorous volunteers, using high-throughput sequencing technologies	Healthy men and women (27); age: 25-59 y; BMI: 16-29 kg/m^2^; parallel study; 3 mo	Intervention: Lacto-ovo-vegetarian diet switch from omnivorous, short-term Control 1: Habitual omnivorous diet Control 2: Habitual long-term lacto-ovo-vegetarian diet	No changes in α- diversity, gene count between start and endpoint of the study ↓ β-Diversity (trend in Jensen-Shannon divergence) after short-term vegetarian diet (intervention)	Short-term animal-based diet/intervention group (as compared with baseline): ↑ *Alistipes* Short-term vegetarian diet/intervention group: ↓ *Alistipes* sp (unclassified *Alistipes* sp HGB5, *A shahii, A putredinis*) Omnivorous: ↑ *Bacteroides finegoldii*; Long-term vegetarians: ↑ *Peptoniphilus duerdenii*, *Clostridium symbiosum*, *Blautia hydrogenotrophica* (formerly, *Ruminococcus hydrogenotrophicus*) Change of diet from omnivorous to lacto-ovo-vegetarian: ↑ *Roseburia inulinivorans*, *Ruminococcus lactaris*, *Lactiplantibacillus plantarum* (formerly, *Lactobacillus plantarum*) ↓ *Streptococcus thermophilus* or *Proteus mirabilis*
Zou et al,^31^ 2020, China	To understand the effects of short-term CR diet on the gut microbial community and amino acid metabolism in nonobese adult individuals	Nonobese healthy individuals (41: M = 17, F = 24); age: 30±6 y; BMI <28 kg/m^2^; uncontrolled longitudinal study with all volunteers receiving the same intervention but with no control group; approximately 4 wk	CR diet: ∼60% calories of the recommended daily calorie intake for M and F in the 2016 Dietary Guidelines for Chinese Residents; M: 2400 kcal/d; F: 2000 kcal/d; average daily calorie supply in this study was 1414.9 kcal/d for M and 1210.6 kcal/d for F, with 43% from CHO, 25% from protein, and 32% from fat Provision of 5 different types of low-calorie meals for the 5-d workweek, with 3 meals/d (breakfast, lunch, and dinner)	Participants divided into 2 enterotypes: No significant changes in α-diversity and β-diversity at the gene and species level before and after the intervention in the 2 enterotype groups	**—**

a Direction of the association not known.

Abbreviations: BD, baseline diet; BMI, body mass index; CAFE, controlled-feeding experiment; CHO, carbohydrate; ConD, control diet; CR, calorie-restricted; CRD, caloric-restriction intervention diet; F, females; FMD, fiber-enriched Mediterranean diet; HDL-C, high-density-lipoprotein cholesterol; KD, ketogenic diet; LGC, low gene count; M, males; MD, isocaloric Mediterranean diet; MedD, Mediterranean diet; OF, overfeeding; OV, omnivore; PH, planetary health; TG, triglycerides; UF, underfeeding; VV, vegan/vegetarian; WD, Western diet; WMD, weight-maintaining diet; –, negative association; +, positive association; ↑, higher; ↓, lower.

**Table 2.** Details of Observational Studies Included in the Review

Study, year, country	Primary objectives	Population (*n*)	Study design, duration	Diet and microbial diversity	Diet and microbial composition
Specific food groups					
Bolte et al,^32^ 2021, The Netherlands	To unravel interactions between diet, gut microbiota, and their functional ability to induce intestinal inflammation	General population and patients with intestinal diseases from northern Netherlands (1450); 4 sub-cohorts, Crohn’s disease (205), ulcerative colitis (126), IBS (223), and healthy controls (871)	Population-based, cross-sectional	—	*Escherichia coli*, *Bacteroides fragilis*, and *Parabacteroides*: (–) bread and legumes *Faecalibacterium prausnitzii*: (+) fruits, red wine and oily fish; (–) high-sugar foods (soft drinks, sweets) *Roseburia hominis*: (+) nuts, oily fish, vegetables, legumes, cereals and plant protein *F prausnitzii*, *Eubacterium hallii*, *Blautia obeum* (formerly, *Ruminococcus obeum*), *Ruminococcus lactaris*, *Anaerostipes hadru*s, and *Alistipes putredinis*: (+) red wine *Bifidobacterium*: (–) red wine *Oscillibacter*: (+) coffee Lactic bacteria: Buttermilk and yogurt^a^ Bacillota (formerly, Firmicutes): (+) total intake of animal protein and fat; (–) plant protein and carbohydrate intake *Bifidobacterium* abundance: (+) plant protein and bread intake; (–) total fat and animal protein intake, cheese, and fish *Bifidobacterium dentium*: (+) meat, animal protein, and butter *Erysipelotrichaceae*, *Ruminococcus* sp (*Blautia* genus), and *Streptococcus* sp: (+) animal protein, (–) plant protein intake *Blautia*, *Lachnospiraceae*, and *Enterocloster bolteae* (formerly, *Clostridium boltae*): (+) fast-food and savory snacks
Le Roy et al,^33^ 2022, UK, The Netherlands, France	To define the link between the gut microbiota and yogurt-associated health benefits	4117 aging twins; MGS (400 yogurt eaters + 144 yogurt non-eaters)	TwinsUK cohort between 1993 and 2015	No significant results	*Streptococcus thermophilus*, *Bifidobacterium animalis*: (+) yogurt consumption
Taylor et al,^34^ 2020, USA	To analyze a subset of the American Gut Project (AGP) cohort based on self-reported consumption of fermented foods (fermented plants); to explore the longitudinal stability and function of the gut microbiota using untargeted high-performance liquid chromatography–tandem mass spectrometry and 16S rRNA amplicon sequencing, as well as shotgun sequencing on a subset of participants at a single time point	Cross-sectional cohort (6811) and targeted 4-wk longitudinal study (115); age: 19-70 y; BMI: 15-50 kg/m^2^	Cross-sectional and targeted 4-wk longitudinal study	—	*Lactobacillus acidophilus*, *Levilactobacillus brevis* (formerly*, Lactobacillus brevis*), *L kefiranofaciens*, *Lentilactobacillus parabuchneri* (formerly*, Lactobacillus parabuchneri*), *L helveticus*, and *Latilactobacillus sakei* subsp *sakei* (formerly*, Lactobacillus sakei*), *Prevotella melaninogenica*, *P multiformis*, *Enorma massiliensis*, *Enterococcus cecorum*, *Bacteroides paurosaccharolyticus*, *Streptococcus dysgalactiae*: Consumers of fermented foods^a^
Zhernakova et al,^35^ 2016, The Netherlands	To understand environment-diet-microbe-host interactions	Sub-cohort of the LifeLines cohort Danish population (M = 474, F = 661; 1135); age: 18-81 y; BMI: 16.7-48.5 kg/m^2^	Population-based prospective cohort studies (sub-cohort)	↑ Diversity: Buttermilk (sour milk with a low-fat content); coffee, tea and red wine ↓ Diversity: High-fat (whole) milk (3.5% fat content), sugar-sweetened soda	*Leuconostoc mesenteroides* and *Lactococcus lactis*: Buttermilk^a^ *F prausnitzii*: Red wine^a^ Bifidobacteria: (+) total carbohydrate intake *Lactobacillus*, *Streptococcus*, and *Roseburia* species: (–) total carbohydrate intake
Diet types or patterns					
De Angelis et al,^36^ 2020, Italy	To show the molecular relationship between diet and metabolic functions of the intestinal microbiome for omnivorous, vegan and vegetarian volunteers; to identify the effects of key dietary components on microbial functions	Generally healthy, Italian adults (30; M = 15, F = 15); age: 25-55 y (36 ± 7.0); BMI >18 (21.89 ± 2.20) kg/m^2^	Subset from a cross-sectional cohort recruited between January and September 2013	No differences	↑ *Lachnospira* abundance: Vegetarians and vegans ↑ *Ruminococcaece* abundance: Omnivores
Kong et al,^37^ 2017, France	To examine the relationship between different dietary patterns, metabolic and inflammatory variables, and gut microbiota	Overweight and obese participants (M = 6, F = 39; 45); lean subjects (14); age: 25-65 y; BMI: ≥25 to <38 kg/m^2^	Prospective, controlled, not randomized trial	Participants in the healthiest dietary pattern cluster: ↑ gene richness and diversity	Total bacterial gene counts: (+) fruits and soups
Shetty et al,^38^ 2022, The Netherlands	To investigate whether different long-term dietary habits result in a gut microbiome composition that deviates from that observed in westernized populations; to identify differences in species-specific contributions to key metabolic pathways	149	—	—	↑Firmicutes/Bacteroidetes ratio: In the pescatarian and vegan group (vs omnivore and vegetarian group); loss of certain VANISH taxa (Succinovibrionaceae, Paraprevotellaceae, Prevotellaceae, and Spirochaetaceae) and overall low abundance of Prevotellaceae: No significant differences between the diet groups BloSSUM taxa: Observed in all diet groups
Stege et al,^39^ 2022, The Netherlands	To check if the long-term dietary habits within a single geographical region impact the human gut resistome in the general Dutch population	149	—	No significant results	↓ *Ruminococcus torques*, *Streptococcus thermophilus*, *Clostridium* sp, *Clostridium phoceensis*, and *Clostridium saccharolyticum*: More in vegans than omnivores *↓ Streptococcus thermophilus*, *Lactococcus lactis*, and *Firmicutes bacterium* CAG:313: In vegans compared than pescatarians *↑ Eubacterium eligens*: In pescatarians more than omnivores *↑ Lactobacillus delbrueckii*, *Coprococcus comes*, *Dorea formicigenerans*, *Dorea longicatena*, *Lawsonibacter asaccharolyticus*, and *Phascolarctobacterium* CAG:266: In omnivores more than vegans
Tarallo et al,^40^ 2022, Italy	To explore the relationship between miRNA profiles and the gut microbiome according to different long-term dietary regimes	120	Cohort; May 2017 and July 2019	—	↑ *Prevotella copri* and *Roseburia* sp CAG 182: VT/VN more than omnivores *↑ Bacteroides dorei* (currently *Phocaeicola dorei*): In omnivores
Wang et al,^41^ 2019, China and USA	To explore the possible links between the gut microbiota and the circulating gut microbiota–host co-metabolites among vegetarians and omnivores	Healthy adults (36), age: 18-55 y	Vegetarian (*n* = 12), a lacto-ovo-vegetarian (*n* = 12), and an omnivorous (*n* = 12) diet for at least 6 mo	No differences with either Shannon-Wiener index or β-diversity	↑ *Prevotella* enterotype: In lacto-ovo-vegetarians and vegans more than omnivores ↓ *Bacteroides* enterotype: In lacto-ovo-vegetarians and vegans more than omnivores ↑ *Alistipes*, *Bacteroides*, *Bilophila*, *Collinsella*, and *Parabacteroides*, ie, *Alistipes putredinis*, *Bacteroides clarus*, *Bacteroides fragilis*, *Bacteroides gallinarum*, *Bacteroides stercoris*, *Bacteroides vulgatus*, *Bilophila wadsworthia*, *Collinsella aerofaciens*, and *Parabacteroides merdae*: In omnivores more than lacto-ovo- vegetarians and vegans ↑ *Prevotella bivia*, *P bryantii*, *P buccae*, and *P copri:* In lacto-ovo-vegetarians and vegans more than omnivores
Nutrient intakes					
Kushulugova et al,^42^ 2018, Kazakhstan	To characterize the gut microbiota of Kazaks and its correlation with clinical (MetS vs healthy) and lifestyle parameters (diet), seasonal effect, influence of NAR synbiotics (6 probiotics + prebiotics, fish collagen and pectin)	Healthy subjects (26) and MetS (58) patients (84); age: 25-75 y	Exploratory	—	Bifidobacterium (*B catenulatum* and *B pseudocatenulatum*): (+) alcohol
Larke et al,^43^ 2023, USA	To characterize the monosaccharide composition of diets in a healthy US adult cohort; followed to assess the relationship between monosaccharide intake, diet quality, characteristics of the gut microbiota, and gastrointestinal inflammation	180	Observational, cross-sectional study; May 2015– July 2019	No significant results	High monosaccharide consumers: *↑ Ruminiclostridium_E* (arabinose)*,* CAG-180 (*Acutalibacteraceae*) (xylose), and *Lachnospira* (GalA) Low monosaccharide intake groups: *↑ Blautia* (arabinose and xylose) and *Faecalitalea* (GalA)
Liu et al,^44^ 2016, China	To characterize the Mongolian gut microbiota; to compare the intestinal microbiome among Mongolian, Han, and European cohorts and to attribute the specific signature of Mongolian gut microbiota to their unique genotype, dietary habits, and living environment	Healthy Mongolian adults (110); Hans (268)	—	—	*Collinsella aerofaciens*: (+) protein, potassium, zinc, iron, and VB1 and species *Bifidobacterium adolescentis*: (+) elements selenium and magnesium
Ma et al,^45^ 2021, USA	To examine the relationship between dietary fiber intake, the gut microbiome, and chronic systemic inflammation	307 generally healthy men aged 70.6 ± 4.3 y	Population based, 2012-2013	—	↑ *Eubacterium eligens*, *Faecalibacterium prausnitzii*, and genus *Roseburia* ↓ *Clostridium, Lachnospiraceae*, and *Ruminococcus* spp: recent and long-term higher dietary fiber ↑ *Haemophilus parainfluenzae* and *Bacteroides cellulosilyticus*: ↑ fiber intake
Dietary indices or scores					
Li et al,^46^ 2021, USA	To investigate the interrelations between hPDI, gut microbiome, and cardiometabolic markers	Healthy men (303); age: 40-71 y; BMI: 25.2 ± 3.6 kg/m^2^	Cross-sectional; 1 y	—	*Bacteroides cellulosilyticus* and *Eubacterium eligens*: (+) hPDI *Ruminococcus torques*, *R gnavus*, *Clostridium leptum*, *Lachnospiraceae bacterium* 1_4_56faa, and *Erysipelotrichaceae* bacterium 21_3: (–) hPDI *B cellulosilyticus*, *E eligens*, *R torques*, *R gnavus*, *Lachnospiraceae* bacterium 1_4_56faa, and *Erysipelotrichaceae* bacterium 21_3: (+) dietary fiber intake *E eligens*, *R torques*, and *Erysipelotrichaceae* bacterium 21_3: (+) fruits *B cellulosilyticus*: (+) whole grains; *Lachnospiraceae* bacterium 1_4_56faa and *Erysipelotrichaceae* bacterium 21_3: (+) frequency of nut consumption
Xiao et al,^47^ 2022, China	To investigate the relation between dietary diversity and the gut environment as well as host metabolism from a multi-omics perspective	1916	Population-based cohorts; GNHS: 2008–2013 and then followed up every 3 y; follow-up visit between 2014 and 2018	—	*↑ Paraprevotella* spp, *Paraprevotella clara*, *Paraprevotella xylaniphila*, and *Oxalobacter formigenes*: Baseline low DDS and stable low DDS groups ↑ *Bacteroides vulgatus* and *Bacteroides ovatus*: stable high DDS groups ↑ *Veillonella atypica* and *Veillonella* spp *Anaerotruncus colihominis*: high DDS group
Yu et al,^48^ 2021, China	To compare the diversity and abundance/presence of fecal microbiome metabolic pathways among individuals according to their long-term diet quality	Generally healthy, older Chinese adults from 2 longitudinal cohorts (144; F = 55%), maintaining a healthy diet (78) and unhealthy diet (66); age: 64 y (mean)	Longitudinal study; (diet data capturing years 1996-2011 and 2002-2011 and stool samples collected during follow-up years 2015-2018)	Healthy-diet group with a small but significant increase in the Shannon index of microbial gene families (vs unhealthy diet group); β-diversity: No differences	—

a Direction of the association not known.

Abbreviations: BloSSUM, Bloom or Selected in Societies of Urbanization/Modernization; BMI, body mass index; DDS, dietary diversity score; F, females; GalA, d-galacturonic acid; hPDI, healthy plant index; IBS, irritable bowel syndrome; M, males; MetS, metabolic syndrome; MGS, metagenomics sequencing; VANISH, volatile and/or associated negatively with industrialized societies of humans; VT/VN, vegetarian/vegan; +, positive association; –, negative association; ↑, higher; ↓, lower.

### Findings of Diet–Gut Microbiota Associations Emerging From Intervention Studies

The study designs and intervention diets were variable, as described in Table 1 and Table S1. The interventions varied in terms of duration, from a couple of days to 6 months.^16–29^ The participants were mostly healthy men and women, with a few studies focusing on groups with overweight/obesity^16^^,^^20–22^^,^^24^^,^^27^ and 1 study involved a mixed group of healthy participants and those with metabolic syndrome.^19^

#### Impacts of Diet Interventions on Gut Microbiota Diversity

The results of the studies revealed that α-diversity had been modulated by a diet intervention only in 4 out of 16 studies. The Shannon index was surprisingly decreased in a trial in which 20 young adults were instructed to consume a high-fiber diet for 3 weeks with an increasing fiber content (40 to 50 g/d).^25^ In 25 volunteers, an increase in Shannon index was detected after underfeeding (50% of the weight-maintaining diet: 20% protein, 30% fat; 50% carbohydrate of daily energy intake) compared with overfeeding (150% of a weight-maintaining diet).^19^ Gene richness was evaluated in 2 studies; it increased after a 6-week energy-restricted, high-protein diet in the participants with overweight and obesity (*n* = 18) with a low microbial gene count^21^ and after an 8-week isocaloric Mediterranean diet in participants with overweight and obesity (*n* = 43).^24^ β-Diversity was affected after a 3-month short-term lacto-ovo-vegetarian diet (*n* = 15), as measured by the Jensen–Shannon distance,^30^ after a 12-week fiber supplement by Bray-Curtis dissimilarity index (*n* = 39; 10 g/d inulin + 10 g/d resistant maltodextrin),^20^ and after 3 weeks of a high-fiber diet by Bray-Curtis distances (*n* = 20).^25^ A 3-week study^23^ where staple carbohydrates—namely, wheat, rice, or oats—were consumed for 1 week in a sequence reported a change in the microbial community composition (Bray-Curtis distances). In the same study, it was observed that wheat had a higher impact, followed by rice and oats on the phylogenetic distance (weighted UniFrac distance). In contrast, there was no difference in β-diversity after either a 10-day high-fiber/low-fat diet or a low-fiber/high-fat diet compared with the control,^29^ in an 8-week study with a whole-grain diet compared with a refined-grain diet,^28^ with an 8-week low-gluten diet compared with a high-gluten diet (no difference in dietary fiber content),^22^ or after a 3-week short-term calorie-restricted diet.^31^

#### Impacts of the Intervention Diets on the Composition of the Gut Bacterial Species

The results from 2 studies indicate that short-term consumption of a calorie-restricted diet,^31^ as well as a whole-grain vs refined-grain diet,^28^ did not modify the gut microbiota at the gene or species level. In contrast, 1 study^30^ in which a diet was changed from an omnivorous to a short-term vegetarian diet resulted in changes in approximately 12 bacterial species. Furthermore, for the control groups, differences in 55 species were observed between omnivorous and vegetarian diets. Compared with the individuals consuming a high-fat diet (4.7 g fiber), those fed the 2-week fiber-enriched Mediterranean diet (54.2 g fiber) increased the abundances of 2 butyrate producer species, *Agathobaculum butyriciproducens* and *Anaerostipes hadrus*.^18^ Another study where the participants consumed a Mediterranean diet for 8 weeks showed evidence of an increased abundance of the fiber-degrading *Faecalibacterium prausnitzii*, as compared with the control group (ie, habitual diet).^24^ In 1 study in which the fiber content of the diet was increased during the 3-week intervention, from 21.0 g per day (±14.2 g/d) to 46.4 g per day (±12.5 g/day), the abundance of *A hadrus* was associated with an elevated fiber intake,^25^ but not when corrected for multiple testing. The abundances of *Bifidobacterium* species as well as butyrate-producing species, *A hadrus* and *Eubacterium hallii*, were found to be reduced in those consuming the low-gluten compared with the high-gluten diet.^22^ One study in which a fiber supplement was used simultaneously with an energy-restricted diet showed an increase in *Bifidobacterium adolescentis* and *Parabacteroides distasonis* as compared with the diet with a placebo supplement.^20^ Underfeeding resulted in an increase in *Akkermansia muciniphila* and 4 *Alistipes* species, as compared with overfeeding.^19^ In another trial, a 4-week ketogenic diet (baseline diet) decreased the relative abundances of several *Bifidobacterium* species.^16^ The abundance of *A muciniphila* was also increased in a Mediterranean diet group of another study in which a Mediterranean diet was combined with weight loss, whereas that of *Lactobacillus ruminis* decreased.^27^ In that same study, a green Mediterranean diet, containing green tea (3–4 cups/day), *Wolffia globosa* (Mankai strain, 100 g/day) and green shake (800 mg/day polyphenols) and weight loss increased the abundances of several species—that is, *Bacteroides massiliensis*, *Paraprevotella clara*, *Alistipes putredinis,* and *Bacteroides vulgatus*. A 3-week trial^23^ in which the staple carbohydrate foods were changed every week altered the gut microbial species. For example, wheat consumption (vs rice and oats consumption) resulted in an increase in 3 species- *Bifidobacterium catenulatum*, *Bifidobacterium bifidum*, and *Alistipes indistinctus*, whereas decreases were observed in *Lactobacillus delbrueckii*, *Ruminococcus gnavus*, *B vulgatus*, and *B massiliensis*. Subsequently, rice consumption reduced the abundances of *Bifidobacterium adolescentis, Bifidobacterium longum*, *Weissella cibaria*, and *Rothia mucilaginosa*; and finally, the oat-rich diet increased the abundances of *B adolescentis*. In a study in which the aim was to quantify and predict individual variations in metabolic responses to standardized meals, it was observed that the abundances of *A hadrus* were associated with healthy plant-based foods and *F prausnitzii* with healthier foods, whereas those of *Clostridium* species were linked to the consumption of less-healthy plant-based (plant-based foods with a higher saturated fat and lower fiber content) and animal-based foods.^17^ One study^26^ that observed relations between the omnivorous, vegan/vegetarian, and planetary health diet and the gut microbiota also reported an increase in the abundances of the species of *Bifidobacterium*, *Prevotella*, and *Gemmiger* (for vegan/vegetarian diet at baseline vs omnivorous diet). Changing the diet to a planetary health diet showed an increase in the abundances of *Bacteroides* species, while it lowered the abundances of Firmicutes species (Table 1).

#### Impact of Intervention Diets on Functional Potential of the Gut Microbiota

Of all the 16 intervention studies examined here, 12 analyzed the predicted function profile and, of those, 9 reported statistically significant results (Table S2). In 1 study,^30^ many Kyoto Encyclopedia of Genes and Genomes (KEGG) orthologous modules were associated with the consumption of both a short-term and a long-term vegetarian diet as well as an omnivorous diet. In the participants consuming the omnivorous diet, enrichment of the module of the osmoprotectant transport system, mediating the cellular uptake of choline, carnitine, and betaine (linked to meat intake), and modules of type II and IV general secretion systems were associated with cholera toxin and intracellular toxins. In both short- and long-term vegetarians, pyruvate:ferredoxin oxidoreductase, which is involved in the production of SCFAs, was enriched. A calorie-restricted diet with a fiber supplement evoked more changes in the functional profile than that of a similar calorie-restricted diet but with a placebo instead of fiber.^20^ Other investigators evaluating the effect of fiber on the functions of the gut microbiota have claimed that a high-fiber vs a high-fat diet led to distinct differences in the bacterial secretion system, protein export, and lipoic acid metabolism.^29^ Another group reported an increase in the d-lactose/l-arabinose transport system substrate-binding protein and a glycosyl-1-phosphate transferase in individuals consuming a whole-grain diet,^28^ but this result was not verified in another study.^25^ Three groups found that a Mediterranean diet influenced the functional profile (eg, enrichment of the genes related to carbohydrate degradation which are linked to butyrate metabolism)^24^ and resulted in an increase in 2 sulfate degradation pathways and a decrease in the oxidative phase of the pentose phosphate pathway^27^ as well as alterations in as many as 27 different metabolic pathways.^18^ It is noteworthy that in 1 of the studies, a lifestyle intervention (physical activity) was implemented along with 2 Mediterranean diet groups (with and without *Wolffia globosa*, an aquatic plant) and a control group, and this phase lasted 6 months.^27^ In the Mediterranean diet intervention studies, the duration of the dietary intervention was only 2 weeks^18^ or 8 weeks^24^ without any changes in other lifestyle parameters. When compared with a high-gluten diet (18 g gluten), a low-gluten diet (2 g gluten) changed 88 KEGG orthologs and 38 KEGG modules; for example, the abundances of the modules related to carbohydrate metabolism (eg, arabinose degradation) and uptake (eg, the l-arabinose/lactose transport system) were decreased in the individuals consuming the low-gluten diet in comparison to those consuming the high-gluten diet.^22^ The authors suggested that a low-gluten diet had affected bacterial carbohydrate degradation, which may have been attributed to the differing arabinose content in these 2 diets.^22^ The study with a change in the staple carbohydrate food^23^ reported that there was a reduction in microbial biosynthesis of branched-chain amino acids following wheat consumption, whereas more extensive changes occurred in fructose metabolism and glycolysis as the diet changed from wheat to rice and finally to oats.

#### Summary of the Evidence From Diet Intervention Studies

Most intervention studies evaluated the effect of fiber on the composition and function of the gut microbiota. It seems that fiber intake is associated with an increase in the Shannon index and in the abundances of beneficial fiber-degrading and butyrate-producing bacteria species as well as the activation of certain pathways—for example, those related to the bacterial secretion system, protein export and lipoic acid metabolism, and the lactose/l-arabinose transport system substrate-binding protein. Most of the studies evaluated differing aspects of the diet, and the results were variable; thus, it is rather difficult to draw any comprehensive conclusions on how the different aspects of diet interact with the gut microbiota.

### Findings of Diet–Gut Microbiota Associations in Observational Studies

The reviewed observational studies included both cross-sectional and longitudinal study designs, with diet being evaluated in several ways, including dietary patterns, foods or food groups (eg, cereals, fruits, vegetables), and nutrients in relation to the composition, diversity, and predicted functions of the gut microbiota (Table 2).

#### Diet and Gut Microbiota Diversity

In the reviewed studies, a higher α-diversity (gene richness and diversity,^37^ Shannon index^35^^,^^48^) was found to be associated with the recommended healthy diet patterns. The remaining reviewed studies^32–34^^,^^36^^,^^38–47^ reported no association of foods, food groups, or diet patterns with either α- or β-diversity.

#### Diet and Gut Microbiota Composition

The results of diet–gut microbiota relations at the species level are shown in Table 2. The key findings include an observation that higher scores on a plant-based diet index (healthy plant-based diet index [hPDI]^38^; details in Table S1) were associated with higher abundances of *Bacteroides cellulosilyticus* (cellulolytic bacterium) and *Eubacterium eligens* (pectin-degrading, butyrate producer), and lower abundances of *Ruminococcus torques*, *R gnavus* (mucolytic species), and *Clostridium leptum* and *Lachnospiraceae* sp (SCFA producers). Another study^47^ examined the relationship with the dietary diversity score (DDS) (Table S1) and observed that a lower score was associated with higher abundances of *Paraprevotella clara*, *Paraprevotella xylaniphila* (acetate producers), and *Oxalobacter formigenes* (formate producer), whereas a higher score with a higher abundance of *B vulgatus*, *Anaerotruncus colihominis* (butyrate-producing bacteria). When different dietary types (lacto-ovo-vegetarian/vegetarian/vegan, omnivore, or pescatarian) were examined, abundances of the bacteria differed in the diets compared. For example, as compared with omnivores, the higher abundances of *Lachnospira* species (SCFA producers; vegetarians and vegans)^36^ and 4 *Prevotella* species (lacto-ovo-vegetarians and vegans),^40^^,^^41^ and the lower abundances of *R torques* (mucolytic bacterium), *Streptococcus thermophilus*, *Clostridium phoceensis*, and *Clostridium saccharolyticum* (vegans), and *E eligens* (pescatarians),^39^ were detected in these different vegetarian diets. However, as compared with the different vegetarian diets, in omnivores higher abundances of some of the butyrate-producing bacteria,^36^^,^^39^^,^^40^ and a few bile-tolerant bacteria such as *Alistipes* and *Bilophila*,^41^ were reported. A higher fiber intake in these observational studies was positively associated with the abundances of *B cellulosilyticus, E eligens,*^45^^,^^46^  *R torques*, *R gnavus*,^46^ and *F prausnitzii* (butyrate producer),^45^ and negatively with abundances of the species of *Clostridium*, *Lachnospiraceae*, and *Ruminococcus*.^45^

#### Diet and Gut Microbiota Functional Potential

Twelve of 17 observational studies (with the exception of references ^34^^,^^35^^,^^37^^,^^39^^,^^42^) reported the predicted functions of the gut microbiota (Table S3). Overall, a healthy diet pattern was positively associated with the pathways related to cofactor, carrier, and vitamin biosynthesis and the tricarboxylic acid (TCA) cycle, and negatively with pathways related to the biosynthesis of certain sugars, sugar nucleotides, amines, and aromatic compounds.^48^ Another study^47^ that used a diet diversity score reported a positive association between a high DDS and pathways involved in bacterial urea cycle function and amino acid biosynthesis. One study^46^ reported that higher hPDI scores were positively associated with the pathways for branched-chain amino acid biosynthesis and fermentation. When considering certain specific types of diets (vegetarians or omnivores), SCFA metabolism–related pathways were different in vegetarians/vegans (butyrate production from pyruvate/acetyl-CoA) in comparison to omnivores (butyrate production from amino acids). Additionally, nitrogen metabolism pathways were enriched more in vegan/vegetarians than in omnivores.^36^ There is also a report describing an enrichment of the pathways related to fatty acid degradation, butanoate metabolism, tyrosine metabolism, branched-chain amino acid degradation, and xenobiotic degradation pathways processing aromatic compounds (eg, chloroalkane) in vegans/vegetarians when compared with omnivores.^41^ Similarly, 1 study^40^ observed a positive relationship of the pathways related to the biosynthesis of amino acids, biogenic amines, or their precursors and the biosynthesis of vitamins in omnivores. Interestingly, only 1 study^38^ considered differences in the species-specific contribution to the gut metabolic modules between diet groups (vegans, vegetarians, omnivores, and pescatarians) (Table S3), and reported differences in the contribution of 9 bacterial species to the amino acid degradation module and 8 species in the carbohydrate degradation module.

#### Summary of the Evidence From Observational Studies

To summarize, diet choices that reflect dietary recommendations (such as a higher consumption of fruits, vegetables, dairy, fish/seafood, nuts, and legumes, and a lower consumption of refined grains and red and processed meat) seem to have positive associations with α- and β-diversity, although an association with β-diversity was not observed in some studies.^48^ Food choices and dietary patterns seem to be associated with the abundances of certain bacterial species as well as with the abundances of the functional pathways related to cofactor, carrier, and vitamin biosynthesis; TCA cycle; as well as fermentation. However, one should also consider the potential functional redundancy of the gut microbiota, where several gut bacteria can contribute to similar functional roles.

## DISCUSSION

In this section, the authors critically discuss the methodological aspects that relate to the design and execution of the studies, the criteria for the selection of study participants, the collection of data, and downstream analyses that may have influenced the interpretation of the results. Furthermore, the authors compare the results obtained by metagenomics sequencing with those obtained by 16S rRNA sequencing for diet–gut microbiota relations, taking dietary fiber as an example.

### Factors Related to Study Design, Execution, and Participants

Randomized controlled trials (RCTs) are the gold standard for studying the effect of a treatment on any outcome variable—in this case, diet on the gut microbiota. A few of the reported interventional studies were not randomized but only had a single-arm or had 1 or more intervention groups but not a control group, which may lower the quality of these study designs.^17^^,^^23^^,^^25^^,^^31^ In the crossover studies, the duration of the washout period ranged from 2 weeks^18^ to 6 weeks,^22^^,^^28^ but in most of the cases this duration had not been justified. The appropriate duration of the washout period has not been defined and it is not known if these rather short washout periods are truly sufficient. On the other hand, longer washout periods may increase the total duration of the study and, in turn, can affect the motivation and compliance of the participants.

In terms of randomization, not all review articles mentioned how the allocation into the groups had been made. If one performs randomization, this diminishes the risk of bias arising from the selection of the study population. Furthermore, in general, there is a recruitment bias related to RCTs, as health-conscious and motivated individuals are more likely to enroll, and therefore these studies might lack a target group that would likely benefit most from the intervention.^49^ The choice of a control arm is commonly challenging. In dietary supplement studies, the control is usually a placebo supplement. However, not all of the dietary intervention studies provided a control diet.^17^^,^^23^^,^^25^^,^^31^ In these trials, as well as in the single-arm studies, some variables might have changed regardless of the intervention, and these could have influenced the gut microbiota. In this respect, it is self-evident that the investigators should monitor the background diet, but unfortunately, this is not always done.

**Figure 2.**
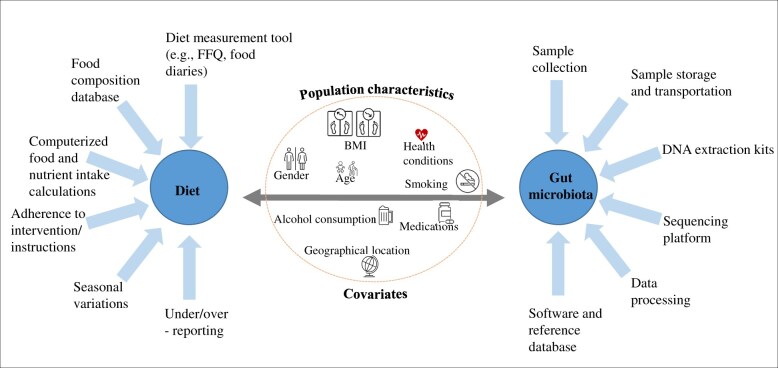
Factors That Affect the Diet and Gut Microbial Data: Potential Covariates in the Studies That Can Influence the Overall Diet–Gut Microbiota Interactions, Related Results, and Interpretations. Abbreviations: BMI, body mass index; FFQ, food-frequency questionnaire

The study participants’ characteristics are factors that may account for the significant differences in the research results reported (eg, age, country of residence, including aspects related to food culture, and body mass index [BMI]). Most of the intervention studies included overweight and/or obese adult participants in addition to normal-weight participants; only 1 study examined only normal-weight participants,^18^ whereas 4 studies did not provide information on BMI.^23^^,^^25^^,^^38^^,^^39^ An increase in both BMI and visceral fat is linked to adverse metabolic effects and altered gut microbiota.^50^^,^^51^ Studies indicate that individuals with obesity may already have different gut microbiota as compared with their normal-weight counterparts.^52^ Therefore, BMI, and optimally, body composition, should be taken into consideration either while planning the study or taken into account in the analyses. For instance, in the studies using fiber-rich diet or foods,^18^^,^^20^^,^^24^^,^^25^^,^^28^^,^^29^ BMI was between 19 and 35 kg/m^2^ (normal-weight to obese) (Table S4), which may also influence the overall conclusions. BMI was included as a covariate in 12 studies,^17^^,^^20^^,^^24^^,^^29–32^^,^^40^^,^^42^^,^^43^^,^^47^^,^^48^ weight in 2 studies,^27^^,^^28^ and fat mass and fat-free mass in 1 trial,^19^ while others included no covariates.^16^^,^^18^^,^^23^^,^^25^^,^^26^^,^^34^^,^^36^^,^^38^^,^^39^^,^^41^^,^^44^ It is of importance that the covariates included must be carefully chosen and justified since the number of covariates influences the statistical power of the analyses. Most intervention and association studies controlled for age and sex (Figure 2). In one of the studies, fiber supplementation showed gender-dependent effects on the gut microbial species.^20^ The reviewed studies were conducted in different geographical regions, which may affect the gut microbiota, possibly due to distinct lifestyle habits or genetic background. Most of the studies defined the study participants as healthy, which was an inclusion criterion for the publications in this review, but some studies nevertheless included participants with conditions such as metabolic syndrome,^42^ impaired glucose tolerance,^19^ or dyslipidemia.^27^ While some studies controlled the analyses for the disease status or medications,^32^^,^^42^^,^^45^^,^^47^ the majority did not take these factors into account.^16–22^^,^^24–29^^,^^33^^,^^34^^,^^37–40^^,^^43^^,^^44^^,^^48^^,^^53^ In the evaluation of the gut microbiota, treatment with oral antibiotics near to sampling should definitely be an exclusion criterion, since antibiotics exert a large impact on the microbiota. This criterion should extend to any other medical treatment with known effects on the gut microbiota (eg, the antidiabetic drug metformin).^54^ It is still poorly understood which medical treatments and drugs influence the gut microbiota; this issue needs to be clarified in the future. The lapse of time when a past antibacterial treatment still exerts effects prior to sampling may also affect the findings. In a recent Estonian cohort study, it was shown that the administration of antibiotics as long ago as 10 years, as well as the number of antibiotic courses, had altered the gut microbiota.^55^ However, it is usually more feasible that individuals receiving antibiotic therapy within the past weeks or months should be excluded rather than those who received these drugs years ago. Overall, the exclusion criteria applied in the articles included in this review are shown in Table S5.

In sum, well-defined inclusion and exclusion criteria can solve many of the problems related to the participants' characteristics within a study, but since the criteria so often differ from study to study, the comparison is challenging.

### Data Analysis

#### Dietary Data

**Figure 3.**
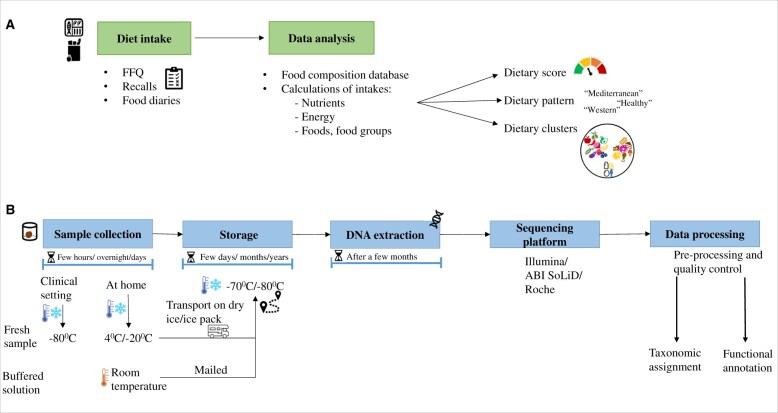
(A) Data Collection and Analysis of the Diet and (B) Steps Involved in Sample Gathering, Data Collection, and Processing of Gut Microbial Composition as Well as the Predicted Functional Pathways According to the Data Described in the Reviewed Articles. Abbreviation: FFQ, food-frequency questionnaire

The steps involved in the collection of dietary data and analysis are shown in Figure 3A. In the reviewed studies, the dietary data-collection methods included food diaries (some using a weighed approach), dietary recalls as well as food-frequency questionnaires (FFQs) (Table S1). Another dietary data collection method available is food-propensity questionnaire (FPQ), although it was not used in the reviewed articles. The food-propensity questionnaire is a qualitative FFQ that assesses both the variation and the frequency of food consumption and is often used together with quantitative methods (eg, 24-hour recall).^56^ Recalls are filled in by the research personnel, which requires good training of the personnel and a reliable memory from the study participant, while food diaries and FFQs are completed by the participants themselves. In all diet recording methods bias may arise from the study participant's behavior (eg, bias related to recall, workload, and social desirability) and can lead to missing data and thus will affect the quality of the data.^49^

Diet quality indices assess the quality of the diet in relation to certain nutrition recommendations or known healthy foods but do not try to make a detailed assessment of nutrient intakes. Some of the studies included in this review used the Healthy Eating Index (HEI),^33^^,^^34^^,^^43^ the hPDI,^46^ the DDS,^47^ and the healthy diet score (HDS),^48^ as shown in Table S1. These types of indices/scores do not always consider energy intake and their development and validation were optimally conducted for the respective study population in that distinct geographical area. Indices specifically developed to depict the dietary quality in reference to that recommended are also available^57^ and may serve as an important tool in gut microbiota studies.^58^^,^^59^ It is important to note that dietary recommendations and food cultures vary from 1 country to the next.^60^ The usage of indices/scores developed in certain countries needs further evaluation, so that those could potentially be applied in other countries or used to compare findings from different locations.^61^

Many dietary data-collection methods are prone to reporting bias. One way to deal with underreporting is to use formulas for the calculation of nutrient intakes that consider underreporting (eg, the Goldberg method).^61^ The compliance to the intervention can be measured, for example, by analyzing the degradation products of the diet^24^ or by using the biomarkers.^28^ It is noteworthy that biomarkers of food intake are not necessarily very specific or sensitive to single foods, and the validation of food-intake biomarkers is an area that requires more research.^62^ In 1 study,^31^ the participants utilized a mobile phone application that monitored their adherence to the diet. If food and nutrient intake analysis could be accurately conducted from images from smart phones, this would be a major step forward, but the research is yet to be conducted.^63^^,^^64^

To conclude, the variability within and between methods may account for the differences in the results emerging from the various studies because of different food groupings, recipes, fortification strategies in each country, calculation systems, real differences in the food content, and missing values in the food-composition database.^65^ Considering these variables in the methodology, it is critical that the dietary intake assessment should be validated for the population of interest.

#### Gut Microbiota Data

The analysis of gut microbiota includes several common steps, as shown in Figure 3B. Details regarding the collection of samples and their transport and storage, subsequent nucleic acid extraction and sequencing methods, followed by the bioinformatics analysis tools applied in the reviewed articles are provided in Table S6.

##### Sample collection, processing, and sequencing methods

Fecal sample collection is a noninvasive method and provides a way to estimate the gut microbiota. Although not convenient, optimally, a fresh sample would be directly subjected to DNA extraction. Typically, in a clinical study setting, a sample is immediately frozen at –20°C or below until DNA extraction and sequencing procedures,^66^ but freezing a fecal sample for storage at –80°C until further analysis is considered to be the gold standard.^67^ In the reviewed studies, the collected fecal samples were frozen either immediately or transferred within a few hours to a household freezer or stored at –20°C and then transferred to −80°C until DNA extraction (Table S6). Only 4 studies reported immediate freezing at either −80°C^29^^,^^30^^,^^47^ or −70°C.^19^ Most often, the samples were collected at home, then transported to the laboratory with cold packs or on dry ice. Two studies^17^^,^^40^ described storing samples with a preservative or a stabilizing solution, and held at room temperature before they were transported to the laboratory for storage at –80°C. To avoid artefacts due to degradation and changes in the microbial composition, it is essential to maintain the ambient temperature and humidity conditions, with minimal fluctuations while the sample is in transit. Only 2 studies^18^^,^^43^ mentioned homogenization of the fecal samples before processing. The entire fecal sample should first be homogenized, sub-sampled, and then used for DNA extraction, as this can overcome a sub-sampling bias (ie, a heterogeneous distribution of microbiota in a fecal sample).^68^^,^^69^

Researchers have demonstrated that the choice of DNA extraction method may affect the overall quantity, purity, and integrity of DNA and subsequently the assessment of the microbial composition (ie, relative abundances of microbial taxa), and have advised that these factors should be considered when comparing the results between studies.^70^^,^^71^

For the identification and characterization of microbiota, 16S rRNA sequencing and metagenomics are the 2 most commonly used methods. Although metagenomics is the optimal choice in gut microbiota studies, it can be expensive for handling larger cohorts,^72^ and the downstream processing of metagenomics reads can be computationally extensive. All of these processes require specific expertise and resources. Additionally, standardized metagenomics data analysis pipelines and comprehensive reference databases are still in progress.

Here, the focus was to include the studies that either used metagenomics solely or applied metagenomics to confirm 16S rRNA findings. The authors made this choice due to the fact that more accurate information is obtained when using metagenomics: metagenomics can achieve taxonomic identification at the species and strain levels^15^^,^^73^ and provides more comprehensive prediction of the functional potential of the microbiota as compared with 16S rRNA. For example, when investigating, eg, gut microbiota diversities, 16S rRNA may use only genus-level information, while metagenomics analysis is based on species-level information. Further, in terms of relative abundances, there are several older studies demonstrating the impact of dietary fiber on gut microbiota using the 16S rRNA method and providing information mostly at the genus level (reviewed, eg, in Swanson et al^74^). When reviewing the recent studies utilizing 16S rRNA for evaluation of how dietary fiber might modify the gut microbiota, 1 study showed compositional changes at the genus level (eg, *Bifidobacterium* following polydextrose supplementation) but not at the species level.^75^ Another study in adults with obesity also reported genus-level modification, including an increase in *Bifidobacterium* after supplementation with inulin-type fructans and an increase in *Anaerostipes* and *Catenibacterium* and a decrease in *Actinomyces* and in the family Erysipelotrichaceae (UCG003) after supplementation with a prebiotic.^76^ The lack of detection of species-level differences could be due to the inability of targeted 16S sub-regions (such as V1–V3) to capture sequence variation in closely related microbial taxa.^77^ Interestingly, similar findings with 16S rRNA^78^ and metagenomics approaches have also been obtained. For example, changes in certain *Bifidobacterium* species or species of other genera, including *Alistipes shahii* and *A hadrus*, were reported in response to dietary fiber consumption (chicory long‐chain inulin consumption) (Table 1). Fundamentally, the species-level information obtained by metagenomics sequencing is more reliable than that obtained by 16S rRNA; in metagenomics, the species are directly identified, while 16S rRNA uses bioinformatics pipelines to predict this information. In some studies, the analytical challenge has been resolved by using 16S rRNA sequencing as a main sequencing method and then metagenomics is applied on a smaller subset for confirmatory analysis.^40^ All in all, due to the differences in the sequencing methods, these 2 approaches are not comparable, both regarding diversity and in areas where higher taxonomic resolution is needed (eg, when investigating how diet influences gut microbial species). Interestingly, few investigators have applied the shallow metagenomics approach to study the relationship between diet and the gut microbiota.^79–81^ Shallow metagenomic sequencing, a method of sequencing DNA at a shallower depth than deep metagenomics, is a middle-ground solution between 16S rRNA and deep metagenomics. However, it is important to note that sequencing depth can affect the sequence read assignment,^82^ and can be considered as a potential confounding factor in gut microbiota studies. Of the selected articles, only 3 studies^32^^,^^35^^,^^48^ included the sequencing depth as a covariate. Also, the use of differing sequencing platforms can introduce variance into the results obtained.^83^ The sequencing platforms used, and the sequencing depth applied in the reviewed articles, are listed in Table S6. All in all, it remains for further validation studies to demonstrate which sequencing methodology, and the subsequent bioinformatics pipeline, is the most accurate method for the study question.

Overall, each of the steps mentioned above potentially can introduce bias and error. For the evaluation of the probable error due to sampling and the subsequent handling, it would be a good practice for investigators to provide detailed reports regarding fecal samples (method and time of collection, defecation frequency/day, and bowel transit time), and transport and storage conditions. Additionally, details regarding sequencing should be included (method, platform, and depth).

##### Data analysis

Metagenomics produces a vast amount of data, which are processed and then analyzed to yield the taxonomical and functional annotations of the gut microbiota. Bioinformatics analyses comprise the pre-processing of data including the removal of primers, adapters, low-quality reads and host sequence reads, mapping of sequence reads, followed by taxonomic and functional annotations. Although all of the articles included in this review followed certain general bioinformatics analysis steps, it has to be noted that different software and programs were used (Table S6). While there is no single perfect method/platform/tool, a standardized approach would be an asset to researchers. It should be noted that microbial taxonomical and functional data are high-dimensional (ie, they contain numerous variables). The choice of analytical and visualization tools depends on the research questions and the type of metadata (clinical and confounding variables), which can affect the interpretations. It is crucial to have a detailed and clear reporting of data analysis steps, parameters chosen, and analytical environment. Checklists such as STORM (Strengthening The Organization and Reporting of Microbiome Studies)^84^ can be helpful. These practices can assure both the reliability and reproducibility of the results, which, in turn, supports open data science. One noteworthy limitation for sharing and making data open access is need for the consent of the participants and data protection of the participants, which is a particular issue in clinical trials. In order to preserve the anonymity of the study participants, it is not always possible to openly share metadata. Hence, such data are either available with restricted access or not available at all. However, code sharing related to data processing and analyses should still be possible. In summary, if researchers are to gain accurate insights, standardized methods are needed to control biological and technical variations and assure the reliability and reproducibility of the results described in the various studies. Also, the application of a multi-omics approach (ie, metagenomics, metabolomics, proteomics, etc) in future studies can provide new insights regarding the diet–gut microbiota relations.

## CONCLUSION

The reviewed literature on diet–microbiota relations indicated that the metagenomics approach yields cumulative evidence that the intake of dietary fiber influences the gut microbiota; this is particularly true from the conclusions described in the intervention studies. This is observed as an increase in the abundances of beneficial fiber-degrading and butyrate-producing bacteria as well as modulations in the functional potential of the microbiota. It is noteworthy that both the intervention and observational studies reviewed here were heterogeneous in their design; selection criteria of the participants; methods and practices of the sample collection; processing, handling, and transport processes; as well as the ways in which the data were analyzed. Metagenomics is becoming more popular, with increasing numbers of recent scientific publications. Thus, it is anticipated that there soon will exist much more research-based evidence on diet–microbiota relationships in healthy human participants, including studies that apply multi-omics approaches to better understand these relationships. For example, a recent review discussed the use and application of several ’omics methods in investigating diet–microbiota metabolism and cardiometabolic health.^85^ One of the fields where further insight is needed is dietary fat–gut microbiota relations. Contrary to dietary fiber, the role of gut microbiota in mediating the impacts of dietary fat on health is less well characterized.^86^ In addition, the diet–gut microbiota relationships in diseased populations also need to be examined in future intervention or observational studies, as these may differ as shown by the research in women with gestational diabetes mellitus (GDM).^87^ The women with GDM did not show changes related to the diet intervention (fish oil and/or probiotics), while in healthy women, modulation of the gut microbiota was seen. Finally, there is a need for more well-designed, conducted, and reported studies, which include standardizing analytics in various phases of the studies, so that researchers can draw more reliable conclusions on diet–gut microbiota relationships.

## Supplementary Material

nuae192_Supplementary_Data
