# Supplementary material for: Diet–Gut Microbiota Relations: Critical Appraisal of Evidence From Studies Using Metagenomics
Source: Nutr Rev. 2024 Dec 24;83(7):e1917–38. doi: 10.1093/nutrit/nuae192 (PMC12166168; doi:10.1093/nutrit/nuae192)
Supplement: nuae192_Supplementary_Data [file nuae192_supplementary_data.docx]

**Supporting Information**

**Table S1. Details about the dietary data collection and assessment in the reviewed articles**

| **Study** | **Dietary data collection** | |
| --- | --- | --- |
|  | **Diet measurement** | **Diet assessment** |
| Ang et al. 2020^1^ | FFQ | **BD** (4-week) (50% CHO, 15% protein, 35% fat) followed by **KD** (4-week) (5% CHO, 15% protein, 80% fat) |
| Asnicar et al. 2021^2^ | FFQ; test meals | At **baseline** (Day 1), participants arrived fasted and were given a standardized metabolic challenge meal for breakfast (0h; 86g CHO, 53g fat) and lunch (4h; 71g CHO, 22g fat);  During the **home-phase** (Days 2–14), participants consumed standardized test meals in duplicate varying in sequence and in macronutrient composition;  To monitor compliance, all test meals consumed by participants were logged in the Zoe app (with an accompanying picture) and reviewed in real-time by the study nutritionists. Only test meals that were consumed according to the standardized meal protocol (outlined in Berry et al. 2020) were included in the analysis. |
| Barber et al. 2021^3^ | Designed diet | **High-residue FMD**: 19% fat, 62% CHO and 16% proteins with 54.2 g fiber.  **High-fat WD**: 51% fat, 27% CHO and 21% proteins with 4.7 g fiber.  **Washout diet**: (23% fat, 55% CHO, 22% proteins) administered two weeks before the high-residue and the high-fat diets |
| Basolo et al. 2020^4^ | Food menu | **WMD**: 20% kcal protein; 30% kcal fat; 50% kcal CHO, adjusted to maintain a stable weight (±1%);  3-d each of 150% of WMD (**OF diet**) and 50% of WMD (**UF diet**) with a 3-d washout period in between in random order in a cross-over design. |
| Benítez-Páez et al. 2021^5^ | 3-day weighted dietary record | **Intervention**: **CRD plus fiber supplement** consisting of 10 g day^−1^ inulin (Fibruline Instant, from chicory and average polymerization degree ≈10) +10 g day^−1^ resistant-maltodextrin (Fibersol - 2, from corn),  **CRD plus placebo** (maltodextrin, isocaloric content) supplement. The fiber and placebo supplement were added to 200 mL of semi-skimmed milk; provided twice per day (morning and afternoon).  **Control**: placebo supplement product was designed to achieve the same caloric content, taste and appearance as the fiber supplement product (CAPSA FOOD, Granda-Asturias, Spain). |
| Bolte et al. 2021^6^ | Semiquantitative FFQ | 173 dietary factors, use of dietary patterns, specific foods and macronutrients; Dutch Food Composition database (NEVO); specific food items were aggregated into 25 food groups in grams per day; In order to identify stable dietary patterns, unsupervised hierarchical clustering of the dietary intake data (gram/day) based on squared Euclidean distances. |
| Cotillard et al. 2013^7^ | 7-day dietary record; interview | 6-week energy-restricted high-protein diet followed by a 6-week weight-maintenance diet;  **first 6-week phase**: energy-restricted high-protein diet (1,200 kilocalories (kcal) per day for women and 1,500 kcal for men: 35% proteins, 25% lipids, 44% carbohydrates) with low glycaemic index carbohydrates and enrichment with soluble fibers;  **second 6-week:** body weight stabilization period with 20% increase in total energy intake, above resting energy metabolic rate of subjects' |
| De Angelis et al. 2020^8^ | 7-day weighed food diary, FFQ | self-declared omnivorous, vegetarian, and vegan diets |
| Hansen et al. 2018^9^ | 4-day pre-coded dietary record; study diary; interview | Low-gluten/high-gluten diet  **Low-gluten intake**: 2g/day  **High-gluten intake**: 18g/day  **Habitual gluten intake**: 12g/day |
| Kong et al. 2017^10^ | 7-day (overweight and obese) and 3-day (lean) unweighted dietary record | PROFILE DOSSIER X029 (Audit Conseil en Informatique Me´dicale, Bourges, France), which has a food composition database initially made up of 400 food items representative of the French diet; Mean food and nutrient intakes; Information on consumed foods could not be directly extrapolated from the program and were therefore coded manually into 26 food groups. |
| Kushugulova et al. 2018^11^ | FFQ (habitual food) | Absolute nutrient amounts per day |
| Larke et al. 2023^12^ | 24-h recall | **HEI** 2015; Food and Nutrition Database for Dietary Studies (FNDDS) versions 4.1 and 2011–2012; monosaccharide composition: Davis Food Glycopedia, that consists of 913 foods across 9 food groups with absolute quantities of 10 monosaccharides: D-glucose, D-galactose, D-fructose, L-arabinose, D-xylose, D-mannose, L-rhamnose, L-fucose, D-ribose, and D-galacturonic acid (GalA) |
| Le Roy et al. 2022^13^ | FFQ | 13 components calculated based on the 131 FFQs entry used to construct the HEI were used to identify association between yoghurt consumption and eating patterns; Twins reporting 'Never or less than once/month' were considered non-consumers, twins reporting frequencies of ‘once a week’ to ‘2-3 per day’ were considered consumers. Twins reporting ‘1-3 per month’ or from ‘4-5 per day’ to ‘6+per day’ were not included. |
| Li J et al. 2021^14^ | FFQ | Designed recipes |
| Li Y et al. 2021^15^ | Semiquantitative FFQ | **hPDI** (Healthy plant-based diet index); whole grains, fruits, vegetables, nuts, legumes, vegetable oils, and tea/coffee as healthy plant food groups; fruit juices, sugar-sweetened beverages, refined grains, potatoes, and sweets/desserts were considered unhealthy plant food groups. Animal food groups included animal fats, dairy, eggs, fish/seafood, meat (poultry and red meat), and miscellaneous animal-based foods |
| Liu W et al. 2016^16^ | FFQ (habitual food) | transformed to the nutrition information; Protein(g), Fat(g), Carbohydrate(g), vitamins: A(ug), C(mg), B1(mg), B2(mg) Nicotinic acid(mg); Trace elements: Mg(mg), Ca(mg), Fe(mg), Zn(mg), Cu(mg), Mn(mg), K(mg), P(mg), Se(ug) |
| Ma W et al. 2021^17^ | FFQ | Participants measured and reported gram weights for foods using a Primo Multifunction Kitchen Scale and ruler before and after eating, provided recipes of home-prepared foods, and returned labels of store-brand products. The Nutrition Data System for Research was used to derive over 150 nutrients and dietary constituents including dietary fiber intake; Daily intake of each nutrient was calculated by multiplying the reported frequency of each food item by its nutrient content and summing across foods, followed by curator quality control. Fiber intake was calculated using the Associations of Official Analytical Chemists method. |
| Meslier et al. 2020^18^ | Food diary | **MedD** (individually tailored diet): fruit and vegetables and nuts (at least 5 portions, ~500 g/day) and nuts (30 g/day) as well as wholegrain products (at least 2 portions, ~200 g/day between wholegrain pasta, bread and breakfast cereal); fish and legumes (at least 2 portions, ~300 g/week of fish and 3 portions, ~300 g/week of legumes); extra-virgin olive oil.  **ConD** (habitual diet): refined cereal products; meat, eggs and dairy products; butter/margarine |
| Oliver et al. 2021^19^ | Normal diet, prepared meals | **Normal diets**: tracking all nutritional information using the smartphone application MyFitnessPal (MyFitnessPal, Inc.);  10 meals per week, with; 15 g of fiber and; 5.8 unique fruits or vegetables per meal, from the food delivery service Thistle (San Francisco, CA, USA). |
| Rehner et al. 2023^20^ | Printed food diary | **Intervention group**: Planetary health diet (details provided online, <https://www.wwf.de/fileadmin/fm-wwf/Publikationen-PDF/Landwirtschaft/wwf-wochenmenue-besseresser-innen-flexitarisch.pdf>);  **Control diet**: vegan/vegetarian diet; and omnivorous diet (details not specified) |
| Rinott et al. 2021^21^ | FFQ, session attendance | 1:1:1 to (1) healthy dietary guidelines, (2) Mediterranean diet, and (3) green-Mediterranean diet weight-loss groups; **iso-caloric Mediterranean group**: consumed 28g/day walnuts (+440mg/d polyphenols provided). **The green-Mediterranean diet**: consumed green tea (3-4 cups/day) and a *Wolffia globosa* (Mankai strain;100g/day) green shake (+800mg/day polyphenols) |
| Roager et al. 2019^22^ | Food diary | Whole-grain intake during-  **whole-grain diet period**: 179±50 g/day  **refined-grain diet period**: 13±10 g/day |
| Shetty et al. 2022^23^ | Not specified | Details not specified |
| Stege et al. 2022^24^ | Not specified | Diet for the past 6 months or more  **Omnivorous diet**: consumption of meat at least three times per week;  **Pescatarian diet**: consumption of fish and animal derived products, but not meat;  **Vegetarian diet**: consumption of animal derived products but not meat or fish;  **Vegan diet**: no consumption of meat, fish, or animal derived products. |
| Tarallo et al. 2022^25^ | FFQ (habitual food) | FFQ consisted of 248 questions concerning 188 different food items and included photos with two or three sample dishes of definite sizes or references to standard portion sizes. The composition in nutrients of individual food items was obtained from Italian food composition tables and the average daily intake of macronutrients and micronutrients for each volunteer was estimated. |
| Taylor et al. 2020^26^ | FFQ | HEI 2010; **Total CLA** (conjugated linoleic acid) consumption (grams/day) obtained from: beef and other meat such as fish and turkey, full-fat dairy products (e.g., milk, butter, cheese, and yogurt), and eggs.  **Total LA** consumption (grams/day) obtained from vegetable oil (e.g., canola and olive), salad dressings containing vegetable oils, butter, eggs, meat (beef, chicken, turkey, and pork), potatoes (e.g., French fries/fried white potatoes, and potato chips), nuts, nut butters and seeds, mixed Mexican dishes, and meat dishes such as stews and casseroles. |
| Wang et al. 2019^27^ | 3 day dietary record | **Lacto-ovo vegetarian diet**: no consumption of meat in any form, but included eggs and dairy products;  **Vegan diet**: no consumption of any animal products |
| Wu et al. 2011^28^ | FFQ; recall | **CAFE**: either a high fiber/low fat diet or a low fiber/high fat Diet;  Total calories in the high fat: 38% from fat, 35% from carbohydrates, and 27% from protein;  Total calories in the low fat: 13% from fat, 69% from carbohydrates, and 18% from protein. |
| Xiao C et al. 2022^29^ | FFQ | To calculate **DDS** (dietary diversity score), the food items were aggregated into 6 major food groups: grains, vegetables, fruits, dairy and dairy products, legumes and legume products, and meat and alternatives. Score 1: If ≥2 servings of a particular food group consumed per week, otherwise, the score for that food group was 0.The total DDS was calculated by summing the scores of all 6 major food groups, ranging from 0 (low dietary diversity) to 6 (high dietary diversity). |
| Yu et al. 2021^30^ | Semiquantitative FFQ (habitual diet); 24 hour recall monthly for over a year | Calculated the **HDS** (healthy diet score) for each participant at each FFQ and generated a cumulative average HDS reflecting long-term diet quality.  8 food groups with equal weights: fruits, vegetables (excluding potatoes), dairy, fish and seafood, nuts and legumes, refined grains, red meat, and processed meat; The FFQs used in the two cohorts (SWHS and SMHS) were very similar, and contained 77 and 81 common food items, respectively.  Participants who stayed in the highest quintile of HDS at each FFQ were defined as having a long-term healthy diet, whereas participants who stayed in the lowest quintile of HDS at each FFQ were defined as having a long-term unhealthy diet. |
| Zhang et al. 2018^31^ | Not evaluated | **Intervention**: lacto-ovo-vegetarian diet switch from omnivorous, short-term ;  **Control**: Habitual omnivorous diet (control 1); Habitual long-term lacto-ovo-vegetarian diet (control 2) |
| Zhernakova et al. 2016^32^ | FFQ | 78 dietary factors |
| Zou et al. 2020^33^ | Standardized diet: digital scale  Non-standardized diet: Picture of food, record, follow up on daily calorie intake with Boohee app | **CR diet**: ~60% calories of the recommended daily calorie intake for men and women in the 2016 Dietary Guidelines for Chinese Residents; Men: 2400 kcal/day; Women: 2000 kcal/day; average daily calorie supply in this study was 1414.9 kcal/day for men and 1210.6 kcal/day for women, with 43% calories from carbohydrates, 25% calories from protein, and 32% calories from fat five different types of low-calorie meals were provided for the five-day workweek (from Monday to Friday), each consisted of 3 meals per day (breakfast, lunch, and dinner) |

*Abbreviations*: BD: Baseline diet; CHO: carbohydrates; ConD: control diet; CR: calorie-restricted diet; CRD: caloric restriction intervention diet; FFQ: food frequency questionnaire; FMD: fiber-enriched Mediterranean diet; h: hours; HEI: Healthy Eating Index; KD: ketogenic diet; MD: isocaloric Mediterranean diet; MedD: Mediterranean diet; OF: overfeeding; UF: underfeeding; WD: western diet; WMD: Weight-maintaining diet.

**Table S2. Results of the impact of diet on the microbial predicted functional pathways in reviewed intervention studies**

| **Study** | **Results** |
| --- | --- |
| Ang et al. 2020^1^ | None |
| Asnicar et al. 2021^2^ | None |
| Barber et al. 2021^3^ | NS between gene families for WD and FMD;  β-diversity (calculated from the expression values of metabolic pathways): ↑ in FMD (vs WD);  After FMD intervention: enrichment of 27 metabolic pathways |
| Basolo et al. 2020^4^ | NS abundances of gene family or metabolic pathway between UF and OF |
| Benítez-Páez et al. 2021^5^ | At time point, week 12: total 154 and 17991 coding metagenes (KO gene categories) changed in the placebo and fiber supplemented groups, respectively; **Placebo group**: ↑ KOs for metabolic pathways involving the alanine, aspartate and glutamate metabolism, and quorum sensing(ko02024); genes for vitamin and cofactor biosynthesis such as riboflavin, Coenzyme-A, and menaquinone;  **Fiber group**: ↑ KOs for the biosynthesis of inosine monophosphate, histidine, heme and siroheme group, ornithine, lysine, chorismate, tyrosine, tryptophan, phenylalanine, NAD, polyamines, and fatty acids, vitamin biosynthesis (cobalamin, pantothenate, and pyridoxal), glycosaminoglycan degradation (e.g., heparin sulfate, chondroitin sulfate, keratan sulfate, and dermatan sulfate), sulfate metabolism, O-glycan biosynthesis, GABA biosynthesis and propanoate metabolism; ↓ genes linked to KDO2-lipid A biosynthesis, biosynthesis of phosphatidylethanolamine and biotin/pimeloyl-ACP,and citrate metabolism |
| Cotillard et al. 2013^7^ | None |
| Hansen et al. 2018^9^ | Following the low-gluten diet period compared with the high-gluten diet intervention change in 88 KOs and 37 KEGG modules;  ↓ abundance of modules associated with carbohydrate metabolism and uptake of carbohydrates;  ↓ abundance of modules associated with the transport of nutrients- abundance of modules associated with bacterial transport of glutamate, zinc/manganese and sulphate;  ↑ abundance of modules associated with transport of cysteine and iron was increased |
| Li J et al. 2017^14^ | First week: ↓ microbial biosynthesis of amino acids (including valine, leucine, and isoleucine) in the first week;  changes in microbial capacity for fructose metabolism and glycolysis and when intake changed sequentially from wheat, rice to oat |
| Meslier et al. 2020^18^ | **After 4 weeks**: ↑ amino acid, carbohydrate degradation, triglyceride and glycoprotein degradation and conversion of acetyl-CoA and glutamate (intervention);  **After 8 weeks**: enrichment in glutamate degradation to crotonyl-CoA was maintained in intervention. |
| Oliver et al. 2021^19^ | NS |
| Rehner et al. 2023^20^ | None |
| Rinott et al. 2021^21^ | **During the weight-loss phase**  **Mediterranean group**: change in 64 microbial metabolic pathways; ↑ two sulfate degradation pathways, and ↓ oxidative phase of the pentose phosphate pathway;  **Green Mediterranean group**: change in 47 microbial metabolic pathways; ↑ LPS biosynthesis and type IV secretion system pathways ; ↓ microbial sugar transport pathways |
| Roager et al. 2019^22^ | **Whole-grain**: ↑ lactose/L-arabinose transport system substrate-binding protein and a glycosyl-1-phosphate transferase |
| Wu et al. 2011^28^ | **Intervention**: functional differences in bacterial secretion system, protein export and lipoic acid metabolism between the high-fat and high-fiber groups |
| Zhang et al. 2018^31^ | Similar trends in enrichment and depletions in pathways in short- and long term vegetarian diet (for e.g. Pyruvate:ferredoxin oxidoreductase, M00310);  Long term vegetarian diet: ↓ abundance of LPS related genes |
| Zou et al. 2020^33^ | NS |

*Abbreviations*: CR, calorie- restricted diet; CRD, caloric restriction intervention diet; FMD, fiber-enriched Mediterranean diet; HGC, high gene count; LGC, low gene count; KO, KEGG Orthologies; MD, isocaloric Mediterranean diet; KD, ketogenic diet; OF, overfeeding; UF, underfeeding; WD, western diet; ↑: higher; ↓: lower; NS: non-significant results.

**Table S3. Results of the associations between diet and the microbial predicted functions in the reviewed observational studies**

| **Study** | **Results** |
| --- | --- |
| Bolte et al. 2021^6^ | Breads and legumes, and fish and nuts: ↑ abundance of pathways for the synthesis of acetate and the urea cycle for detoxification of ammonium;  Coffee: heterolactic fermentation and various glycolytic pathways*;  Consumption of fermented dairy like buttermilk and yoghurt: fermentation of pyruvate to butanediol and peptidoglycan synthesis*;  Total intake of plant-derived protein: ↑ abundance of pathways involved in the synthesis of SCFA, thiamin, biotin, flavin, vitamin B6 and L-ornithine, and the degradation of sugar derivates;  Alcohol, sugar and total plant protein intake: ↓ quinone synthesis pathways  Potato consumption: ↑ abundance of starch degrading pathways |
| De Angelis et al. 2020^8^ | Different SCFA metabolism related pathways: vegetarians/vegans (butyrate production from pyruvate/Acetyl CoA); omnivores (butyrate production from amino acids);  Vegan/vegetarian diet (vs omnivore ): ↑ abundance of nitrogen metabolism pathways |
| Kong et al. 2017^10^ | None |
| Kushugulova et al. 2018^11^ | None |
| Larke et al. 2023^12^ | None |
| Le Roy et al. 2022^13^ | Yogurt consumers: ↑ abundance of peptidoglycan biosynthesis IV pathway |
| Li Y et al. 2021^15^ | ↑ hPDI score: ↑ abundance of pathways of branched-chain amino acid (BCAA) biosynthesis (l-isoleucine biosynthesis I and III and l-valine biosynthesis) and fermentation (pyruvate fermentation to isobutanol);  ↓ hPDI score: ↑ abundance of pathways of purine nucleobases degradation pathway, lipid biosynthesis pathway of stearate biosynthesis II as well as amine degradation pathways of allantoin degradation to glyoxylate II and III;  Relative abundance of amino acid biosynthesis pathways: (+) fiber and fruits, (-) meats |
| Liu W et al. 2016^16^ | ↑ Meat and fermentation: ↑ abundance of pathways of amino acid metabolism and energy metabolism functions |
| Ma W et al. 2021^17^ | ↑ Intake of total fiber and pectin: ↑expression of endoglucanase hydrolyzing of (1,4)-beta- D-glucan linkages in cellulose and glycogen phosphorylase |
| Shetty et al. 2022^23^ | Species-specific differences in contribution to gut metabolic modules between diet groups observed;  Carbohydrate degradation module: 8 species;  Amino acid degradation module: 9 species;  Lipid metabolism: NS;  Vegans and vegetareans: NS (amino acid and carbohydrate degradation modules);  Omnivore and vegetarians: *Bacteroides ovatus(*glycoprotein and lipid degradation);  Omnivores (vs vegan): ↑ contribution of *Ruminococcus torques* (acetate and propionate metabolism);  Omnivores (vs vegans and vegetarians): *Streptococcus thermophilus* and *S.thermophilus* CAG236 (lactate metabolism);  Omnivores (vs pescatarians): ↑ contribution of *Flavinofracter plautii* (propionate metabolism)  Pescatarians (vs omnivore): ↑ contribution of *Roseburia hominis*(lactate metabolism) |
| Stege et al. 2022^24^ | None |
| Tarallo et al. 2022^25^ | Omnivores: ↑ pathway abundances of L-isoleucine biosynthesis IV, L-arginine biosynthesis I and II, super-pathway of L-lysine, L-threonine and L-methionine biosynthesis II, putrescine biosynthesis IV, chorismate biosynthesis from 3-dehydroquinate, formyl-tetrahydrofolate biosynthesis, pantothenate and coenzyme A biosynthesis III |
| Taylor et al. 2020^26^ | None |
| Wang et al. 2019^27^ | Vegans and vegetarians (vs omnivores): ↑ abundance of pathways of fatty acid degradation, butanoate metabolism, tyrosine metabolism, valine, leucine, and isoleucine degradation, and xenobiotic degradation pathways processing aromatic compounds, naphthalene, chloroalkane, and chloroalkene |
| Xiao C et al. 2022^29^ | High DDS: ↑ pathway abundances of l-ornithine de novo biosynthesis, urea cycle, l-citrulline biosynthesis, and l-isoleucine biosynthesis II;  Low DDS: ↑ pathway abundances of l-tryptophan biosynthesis, UDP-N acetyl-d-glucosamine biosynthesis I, the superpathway of pyrimidine deoxyribonucleosides degradation, and the NAD salvage pathway II |
| Yu et al. 2021^30^ | Healthy diet: ↓ pathway abundances for biosyntheses of nucleotidesugars (e.g., CMP-legionaminate and dTDP-l-rhamnose), nucleotides(e.g., guanosine and adenosine), and fatty acids;  ↑ pathway abundances for biosyntheses of tetrahydrofolate, acetyl-CoA, l-methionine, and dTDP-N-acetylthomosamine and for degradation of sucrose, 4-aminobutanoate (GABA), methylglyoxal, and sulfate, degradingaromatic compounds (e.g., catechol and toluene);  CMP-legionaminate biosynthesis: (+) refined grains, (-) fruits, and fish/seafood;  Tetrapyrrole biosynthesis: (-) dairy intake |
| Zhernakova et al. 2016^32^ | None |

*Abbreviations*: DDS: dietary diversity score; hPDI: healthy plant index; SCFA: Short-chain Fatty acids; β: beta; (+) positive, (-) negative, ↑: higher, ↓: lower, *: direction of the relationship not known; NS: no significant differences.

**Table S4. Different criteria used to characterize the study participants according to their health status and BMI in the reviewed articles**

| **Subjects** | **Study** | **Subjects description in the study** | **BMI (kg/m^2^)** |
| --- | --- | --- | --- |
| **Healthy** | Asnicar et al. 2021^2^ | Generally healthy adults | Not specified |
|  | Barber et al. 2021^3^ | Healthy volunteers; only men | 19.2-25.5 |
|  | Bolte et al. 2021^6^ | Healthy control | 25.25 (SD 4.06) |
|  | De Angelis et al. 2020^8^ | Apparently healthy volunteers | BMI >18; 21.89 ± 2.20* |
|  | Li Y et al. 2021^15^ | Healthy men | 25.2 ± 3.6 |
|  | Liu W et al. 2016^16^ | Healthy adults | Not specified |
|  | Ma W et al. 2021^17^ | Generally healthy men | 24.1 (SD 3.3) - 26.9 (SD 4.5) |
|  | Rehner et al. 2023^20^ | Healthy volunteers | 19.8-40.1 |
|  | Roager et al. 2019^22^ | Danish adults, at risk of developing metabolic syndrome | 25-35 |
|  | Tarallo et al. 2022^25^ | Healthy volunteers | Omnivore: 23.6±3.9,  Vegetarians: 21.9±2.6,  Vegan: 21.8±2.9 |
|  | Wang et al. 2019^27^ | Healthy adult volunteers | Not specified |
|  | Wu et al. 2011^28^ | Healthy volunteers | 18.5-35 |
|  | Zhang et al. 2018^31^ | Healthy volunteers | 16-29 |
|  | Zou et al. 2020^33^ | Non-obese healthy | BMI < 28; 23.72 ± 2.81 |
| **Overweight/obese** | Ang et al. 2020^1^ | Only men, non-diabetic | 25-35 |
|  | Basolo et al. 2020^4^ | Healthy obese, (deemed healthy, except for impaired glucose tolerance and obesity) | 32.8 ± 8.0 |
|  | Benítez-Páez et al. 2021^5^ | Overweight participants | Fiber intervention group: 32.8 ± 3.9;  Placebo group: 34.3 ± 4.8, |
|  | Cotillard et al. 2013^7^ | Obese or overweight subjects | Baseline: 33.21 ± 0.55;  6 weeks: 31.29 ± 0.56;  12 weeks: 31.39 ± 0.59;  (expressed as means ± s.e.m.) |
|  | Meslier et al. 2020^18^ | Healthy overweight and obese subjects | BMI ≥ 24; 31.1 ± 4.5 |
|  | Rinott et al. 2021^21^ | Abdominally obese or dyslipidemic men | 31.3 (SD 4.09) |
| **Normal (lean), Overweight, obese** | Hansen et al. 2018^9^ | Middle-aged Danish adults | 25-35; and/increased waist circumference (≥ 94 cm for men and ≥ 80 cm for women) |
|  | Kong et al. 2017^10^ | Overweight and obese, but otherwise healthy; normal as control | Overweight-Obese: 25≤BMI<38;  Normal: 18<BMI<25 |
|  | Kushugulova et al. 2018^11^ | Healthy and non-healthy (case group included participants with overweight, diagnosed diabetes and/or hypertension, i.e., with metabolic syndrome) | Healthy: 22.9-23.1;  MetS: 29-29.1 |
|  | Larke et al. 2023^12^ | Healthy US adult cohort | Normal: 18.5-24.99;  Overweight: 25-29.99;  Obese: 30-44 |
|  | Le Roy et al. 2022^13^ | Adult twins | Yogurt consumers: 25.43 ± 0.08;  Non-consumers: 25.22 ± 0.13 |
|  | Taylor et al. 2020^26^ | Shown in supplemental figure of the article: underweight, normal, overweight and obese | 15-50 |
|  | Xiao C et al. 2022^29^ | Middle-aged elderly adults; | 23 ± 3.0 |
|  | Yu et al. 2021^30^ | Generally healthy, older, urban Chinese adults | 23.7 ± 3.2 |
|  | Zhernakova et al. 2016^32^ | Shown in supplemental figure of article | 16.7- 48.5 (mean 25.3) |
| **Not specified** | Li J et al. 2021^14^ |  |  |
|  | Shetty et al. 2022^23^ |  |  |
|  | Stege et al. 2022^24^ |  |  |
|  | Oliver et al. 2021^19^ |  |  |

*Body mass index (BMI) expressed as (mean ± SD), unless specified in the bracket

**Table S5. Overall exclusion criteria for the study participants in the reviewed studies**

| **Study** | **Exclusion criteria not specified** | **Use antibiotics prior or during study** | **GI diseases^1^** | **Cardiac/renal/**  **hepatic or other diseases^2^** | **Use of medicines** | **Outliers in body/blood marker measurements** | **Use of pre- or pro- biotics or supplements** | **Travel outside study country** | **Pregnancy/ nursing women** | **Intense physical activity** | **Additional details** |
| --- | --- | --- | --- | --- | --- | --- | --- | --- | --- | --- | --- |
| Ang et al. 2020^1^ |  |  | X | X | X | X |  |  |  |  |  |
| Asnicar et al. 2021^2^ |  | X | X | X |  | X |  |  | X |  | Ongoing inflammatory disease; cancer in the last three years (excluding skin cancer); capillary glucose level of >12 mmol l^–1^ (or 216 mg dl^–1^), or type 1 diabetes mellitus, or taking medication for type 2 diabetes mellitus; currently experiencing acute clinically diagnosed depression; vegan or experiencing an eating disorder or unwilling to consume foods that are part of the study |
| Barber et al. 2021^3^ |  | X | X |  |  |  | X |  |  |  | Women |
| Basolo et al. 2020^4^ |  | X | X | X |  |  |  |  |  |  |  |
| Benítez-Páez et al. 2021^5^ |  | X | X |  | X |  | X |  | X | X | Surgical treatment of obesity, abdominal surgery, special dietary habits including vegetarians and vegans or a known food allergy of the food components included in the study supplements (e.g. intolerance towards lactose or maltodextrin), weight change of more than 3 kg within 2 months prior to the study, blood donation less than 1 month prior to or during the study and participation in other clinical trials less than 1 month before study start or inability to comply with the procedures required by the study protocol |
| Bolte et al. 2021^6^ | X |  |  |  |  |  |  |  |  |  |  |
| Cotillard et al. 2013^7^ |  | X |  | X |  |  |  |  |  |  | Undergoing chronic treatment or had been involved in weight-loss programs in the preceding 12 months |
| De Angelis et al. 2020^8^ |  | X | X | X |  |  | X |  | X |  | Diet regimen followed for less than a year, age <18 or > 60 |
| Hansen et al. 2018^9^ |  | X | X |  |  |  | X |  |  | X |  |
| Kong et al. 2017^10^ |  | X | X | X |  |  |  |  |  |  |  |
| Kushugulova et al. 2018^11^ |  | X |  |  |  |  |  |  |  |  |  |
| Larke et al. 2023^12^ |  | X |  |  | X |  |  |  |  |  | Recent surgery or hospitalization; medication for a diagnosed chronic illness |
| Le Roy et al. 2022^13^ | X |  |  |  |  |  |  |  |  |  |  |
| Li J et al. 2021^14^ |  | X | X |  |  |  |  |  |  |  |  |
| Li Y et al. 2021^15^ |  |  | X | X |  |  |  |  |  |  | Stroke, cancer, major neurological diseases |
| Liu W et al. 2016^16^ | X |  |  |  |  |  |  |  |  |  |  |
| Ma W et al. 2021^17^ |  |  |  | X |  |  |  |  |  |  | Coronary heart disease, stroke, cancer (except squamous or basal cell skin cancer), or major neurological diseases |
| Meslier et al. 2020^18^ |  | X | X | X |  |  | X |  | X | X |  |
| Oliver et al. 2021^19^ | X |  |  |  |  |  |  |  |  |  |  |
| Rehner et al. 2023^20^ |  | X |  | X |  |  |  |  | X |  | Active smoking; acute and/or chronic diseases |
| Rinott et al. 2021^21^ |  |  |  | X | X | X |  |  | X |  | Participation in other trial; inability for physical activity; pacemaker; |
| Roager et al. 2019^22^ |  | X | X |  | X | X | X |  | X | X | Participation in other trial; alcohol consumption |
| Shetty et al. 2022^23^ | X |  |  |  |  |  |  |  |  |  |  |
| Stege et al. 2022^24^ |  | X |  |  | X |  |  |  |  |  | Use of insulin, proton pump inhibitors or drugs related to cancer treatment and chemotherapy in the past 3 months |
| Tarallo et al. 2022^25^ |  | X | X |  | X |  |  |  | X |  | Not following dietary regimen for more than 1 year; no use of aspirin or other anti-inflammatory drugs in the month prior to the sampling, no evidence of intestinal and other pathologies |
| Taylor et al. 2020^26^ |  | X |  |  |  | X |  |  |  |  |  |
| Wang et al. 2019^27^ |  | X | X |  |  |  |  |  | X |  | Pre-existing chronic diseases such as hypertension, diabetes mellitus, and cancer |
| Wu et al. 2011^28^ |  | X | X | X | X |  | X |  |  |  |  |
| Xiao C et al. 2022^29^ |  | X |  | X |  |  |  |  |  | X | Cancer and type-2 diabetes |
| Yu et al. 2021^30^ |  | X |  | X | X |  |  |  |  |  | No long-term dietary data |
| Zhang et al. 2018^31^ |  | X |  |  |  |  |  |  |  |  |  |
| Zhernakova et al. 2016^32^ | Described elsewhere |  | X |  |  |  |  |  |  |  |  |
| Zou et al. 2020^33^ |  | X | X | X |  |  | X | X |  |  |  |

^1^GI diseases/ conditions: bacterial overgrowth syndrome, celiac disease, constipation, Crohn's disease, Irritable Bowel Syndrome, ulcerative colitis.

^2^Other diseases: allergies, cancer, chronic diseases, cerebrovascular diseases, HIV/AIDS, neurodegenerative diseases, rheumatoid arthritis, T2D.

**Table S6. Details about the gut microbial data collection and analysis in the reviewed articles**

| **Study** | **Gut microbial sample collection and processing** | | | | | **Bioinformatic analysis** | | | **Covariates** | **Multiple correction** |
| --- | --- | --- | --- | --- | --- | --- | --- | --- | --- | --- |
|  | **Sample collection** | **Storage and transport** | **DNA isolation** | **Sequencing platform** | **Sequence reads depth** | **Read mapping** | **Taxonomic assignment** | **Functional annotation** |  |  |
| Ang et al. 2020^1^ | Collected daily in final weeks of diet stage | Immediately frozen at −80 °C | Bead beating and PowerSoil bacterial DNA extraction kit (MoBio) | Illumina HiSeq 2500, PE | Not mentioned | Bowtie2 | MetaPhlAn2 |  | None | FDR<0.05 |
| Asnicar et al. 2021^2^ | Stored at ambient temperature until transport to laboratory | Aliquoted, and stored at −80 °C | PowerSoilPro, FastDNA, ProtocolQ, Zymo | Illumina NovaSeq 600, 300bp PE | Mean 8.8±2.2 Gb/sample | bioBakery suite | MetaPhlAn 3.0 | HUMAnN 2.0 | Age, BMI | FDR<0.2 |
| Barber et al. 2021^3^ | Collected, homogenized and immediately frozen in home freezers at −20 °C | Transported to laboratory in a freezer pack, and stored at −80 °C | Described elsewhere |  | Not mentioned | Bowtie2 (v2.4.2) | MetaPhlAn 2.0 (v2.9.14) | HUMAnN 2.0 (v2.9.0) | None | FDR<0.25 |
| Basolo et al. 2020^4^ | Collected daily | Immediately frozen at −70 °C | ZymoBIOMICS 96 MagBead DNA Kit (Zymo Research) | Illumina Novaseq S2; 100 bp PE | 381.7 Gb pairs/ 95 samples | QIIME (for 16S);  Bowtie2 | DADA2 (for 16S); MetaPhlAn2 | HUMAnN2 v0.11.1 | Baseline fat free mass, fat mass, age, race and gender | FDR<0.05 |
| Benítez-Páez et al. 2021^5^ | Samples collected and delivered a cooler bag with disposable cooling blocks within 3 h | Aliquoted and stored at −80 °C | Bead beating, QIAamp Fast DNA Stool Mini Kit (Qiagen, Germany) | Illumina Novaseq S2; 150 bp PE | ~3.69 Tb raw data |  | mOTUsv2.0 | KEGG | Age, baseline BMI, gender | FDR≤ 0.05 |
| Bolte et al. 2021^6^ | Collect and immediately in freezer | Transported on dry ice and stored at –80 °C | Described elsewhere | Illumina HiSeq | Sequencing depth > 10 million reads | KneadData integrated Bowtie2 tool (V.2.3.4.1) | MetaPhlAn2 (V.2.2) | HUMAnN2 (V.0.10.0). | Age, gender, sequencing depth (food consumption adjusted for calorie intake); also explored influence of BMI, smoking, hypertenshion (use of antihypertensives), diabetes (use of antidiabetics) and hyperlipidaemia (use of statins) | FDR<0.05 |
| Cotillard et al. 2013^7^ | Self-collected in sterile boxes and stored at −20 °C within 4 h | Aliquoted and stored at −80 °C | Described elsewhere | ABI SOLiD; 35bp SE | Average of 76.5 million±36.5 million (mean±SD) /sample;  total= 393Gb | By using corona_lite (v4.0r2.0), IGC | METEOR |  | Age, gender | FDR |
| De Angelis et al. 2020^8^ | Home collected samples in sterile sampling containers immediately stored at 4 °C | Transported to the laboratory within 12 h of collection at a refrigerated temperature; immediately stored at −80 °C | Powersoil DNA kit (MO-BIO, Carlsbad, CA, USA) | Illumina HiSeq 1500; 151 bp SE | 184 Gb sequences, with an average of 6.81 Gb/sample |  |  |  |  | FDR<0.05 |
| Hansen et al. 2018^9^ | Collected and stored at 5 °C for 24 h | Aliquoted and stored at −80 °C | Described elsewhere | Illumina HiSeq 2000, 100bp PE | 3.7 Gbp to 16.8 Gbp/sample | Cutadapt v. 1.8.1 with Python 2.7.10; trimmed reads mapped using BWA-MEM; IGC | BLAST | EggNOG (v3), KEGG | Age, gender, intestinal transit time | FDR<0.05 |
| Kong et al. 2017^10^ | Collected in sterile boxes and placed at −20 °C within 4 h | Treated, aliquoted and stored at −80 °C | Described elsewhere | ABI SOLiD; 35bp SE | average of 76.5±36.5(mean ± sd) million/ sample | corona_lite (v4.0r2.0) | METEOR software | Part of METEOR | Age | None |
| Kushugulova et al. 2018^11^ | Collected and placed at −20 °C immediately | Within 24h at −80 °C | G’NOME kit (BIO 101) | Illumina HiSeq 2500; 100bp PE | Average 2.7±1.1 Gbp of 100 bp/sample | MOCAT pipeline |  |  | Metabolic syndrome status, BMI | FDR< 0.05 |
| Larke et al. 2023^12^ | A stool specimen was collected within 3 days of the second study visit at the end of the 10–14 day dietary recall period | Kept on blue ice and transported to the research center as soon as possible for same-day processing. homogenization of samples with Stomacher paddle blender prior to freezing at −80 °C | ZymoBiomics DNA miniprep kit | Described elsewhere |  |  | Kraken2; aligned to a custom database [release 95 (13.07.2020)] using Sturo | KEGG | Age, sex, BMI | FDR <0.05, BH |
| Le Roy et al. 2022^13^ | At home using mainly dry sarstedt tube | Samples were stored in the refrigerator for 2 days or less prior to their annual clinical visit at St. Thomas’ Hospital. Once the samples arrived with the clinical team, they were stored at −80 °C until further processing | Described elsewhere | Illumina HiSeq 2500 using SBS kit V4 Chemistry, 125 bp PE | Average number of reads of 54 million per sample before quality control | YAMP pipeline | MetaPhlAn2 v. 2.6.0 | HUMAnN2 pipeline, v0.10.0; UniRef90 proteomic database | Age, BMI, sex, HEI and family structure |  |
| Li J et al. 2021^14^ | The fecal samples of each individual in 4 time points (from week 0 to week 3) | Stored at –80 °C | QIAamp^®^ DNA Stool Mini Kit (Qiagen, Hilden, Germany) | Illumina HiSeq2500; 100bp PE | Average of 10.34 gigabases (Gb) of paired-end reads for each sample, total of more than 1 Tb | IDBA-UD | MetaPhlAn2 | KEGG, CAZy, and ARGs |  |  |
| Li Y et al. 2021^15^ | Described elsewhere | Described elsewhere | Nextera XT DNA Library Preparation Kit | Illumina HiSeq, 100bp PE | Sequenced to a target depth of 1–2 Gnt each | bioBakery, KneadData | MetaPhlAn2 | HUMAnN2 | Proximately to each fecal/blood sample collection, including age, total energy intake, physical activity, smoking, alcohol consumption, Bristol categories, use of antibiotics in past year, and consumption of any probiotics (except yogurt) in the past 2 months | FDR <0.05, BH |
| Liu W et al. 2016^16^ | Described elsewhere | Described elsewhere | Described elsewhere | Illumina HiSeq 2500 | Not mentioned | Constructed Mongolian gene catalogue | MetaPhlAn2 |  | None | None |
| Ma W et al. 2021^17^ | Collected in a container with RNAlater at ambient temperature | Shipped overnight to the Broad Institute of MIT and Harvard, and stored immediately in –80 °C freezers until nucleic acid extraction. |  | Illumina HiSeq; 101 nu PE |  | biobakery | MetaPhIAn 2.6.0 | HUMAnN 2.11.0 | Age, recent antibiotic use, and total calorie intake |  |
| Meslier et al. 2020^18^ | Samples at 4 °C and transported to the laboratory within 24 h | Stored at –80 °C | IHMS SOP P7 V2 | Ion Proton, 150 bp | Average of 22.2 ± 1.6 million reads was produced | Metagenomic species pangenome (MSP); IGC | METEOR | In-house FAnToMet pipeline | Age, sex, BMI, energy intake | FDR<0.05 |
| Oliver et al. 2021^19^ | Collected and immediately stored in the freezer | Transported to –20 °C | ZymoBIOMICS 96 DNA kit (product D4309) | Illumina NextSeq 500; 75 bp PE | ~1.3 million paired-end reads (average per sample; total of 144,023,583 reads and an average of 1,425,976 reads/sample |  | MIDAS, IGGsearch;StrainPhlAn | HUMAnN3, CAZyDB.07202017 | None | FDR<0.05 |
| Rehner et al. 2023^20^ |  | Transferred to the laboratory within 24 h and stored at –80 °C until further processing. | ZymoBIOMIC DNA Miniprep Kit | Illumia Hiseq, 150 bp PE | 3 Gb reads per sample | KneadData | MetaPhlAn3 v3.0.13; ChocoPhlAn |  |  |  |
| Rinott et al. 2021^21^ | Collected at study site, immediately frozen to –20 °C for 1–3 days | Transferred to –80 °C |  | Illumina NextSeq 500/550, 14 bp SE | An average depth of 15.4 ± 2.6 million reads per sample (mean ± standard deviation) | BURST pipeline | BURST pipeline, RefSeq for bacteria and reference sequence in CoreBiome’s Venti database | KEGG | Age, gender, baseline weight, initial 6 month weight loss | FDR<0.05 |
| Roager et al. 2019^22^ | Collected and stored at 5 °C for 24 h | Aliquoted and stored at −80 °C | Described elsewhere | Illumina HiSeq, 100bp PE | The sample read counts ranged from 730 Mbp to 16.8 Gbp | Cutadapt v. 1.8.1 with Python 2.7.10; trimmed reads mapped using BWA-MEM; IGC | BLAST | CaZy and KEGG | Age, gender, body weight | FDR<0.05 |
| Shetty et al. 2022^23^ |  |  |  |  |  | Kneaddata | MetaPhlAn3 | HUMAnN3 |  |  |
| Stege et al. 2022^24^ |  | Transported for a maximum of 24 h by regular mail before storage at −80 °C | QIAamp fast DNA stool mini kit | NovaSeq 6000; 150bp PE | 70.7 M ± 11.3 M reads |  | Either mOTUs2 version 2.5.0 or MetaPhlAn3 |  |  | FDR <0.05, BH |
| Tarallo et al. 2022^25^ | At home in nucleic acid collection and transport tubes with RNA/DNA stabilizing solution (Norgen Biotek Corp) | Stored at −80 °C until RNA and DNA extraction | QIAamp DNA stool MiniKit (QIAGEN) | Illumina HiSeq |  |  | MetaPhlAn3 | HUMAnN3 | Age, sex and BMI. | FDR <0.05, BH |
| Taylor et al. 2020^26^ | Described elsewhere (AGP protocol) | Described elsewhere | Described elsewhere (Earth Microbiome Project (EMP) standard protocols ) | Illumina HiSeq 2500; PE | Target depth of ca. 20 million reads per sample | SHOGUN v1.0.6; Bowtie 2 v2.3.4.3 |  |  | None | None |
| Wang et al. 2019^27^ | Collected at home;transferred to the sterile containers using the olypropylene spoon included; specimens were immediately transported to the laboratory on ice, subdivided into three aliquots | Stored at −80 °C within 2 h | Not specified | Illumina HiSeq 4000; 300bp PE | Not mentioned | BLASTP (version 2.2.28+) | NCBI NR database | KEGG | None | None |
| Wu et al. 2011^28^ | Collected daily | Immediately frozen at −80 °C | MoBio PowerSoil kit | 454 Roche | 1.05 × 10^6^ sequence reads total | QIIME, BLAST (MGS) | QIIME | KEGG | BMI, gender, race, total Fats, SFA | FDR≤ 0.25 |
| Xiao C et al. 2022^29^ | Collected at the visit | Frozen at −80 °C until analyzed |  | Illumina HiSeq: 300 bp PE |  | PRINSEQ v0.20.4 | MetaPhlAn2 v2.6.0 | HUMAnN 2.0 | Age, sex, BMI, total energy intake, physical activity, education level, household income, smoking status, alcohol drinking status, use of antihypertensive drugs, and lipid-lowering drugs | FDR<0.25, BH for pathway analysis |
| Yu et al. 2021^30^ | Sample collected in a tube containing 5-mL 95% ethanol and glass beads, stored at room temperature; transported to a research laboratory within 24 h | Aliquots and stored at −80 °C | Qiagen’s DNeasy PowerSoil kit | Illumina HiSeq XTEN, PE | Median of 27.3 million raw reads per sample (range: 20.1 to 47.0 million reads). After quality-trimming and removal of human reads, on average, 27.1 million reads retained/sample (range: 20.1 to 46.9 million reads) | Trimmomatic (v0.39), Bowtie2 (v2.3.5) | HUMAnN2 (v2.8.1) | HUMAnN2 (v2.8.1) | Sequencing depth, sociodemographic (age at stool sample collection, sex, and income), lifestyle (cigarette smoking, alcohol drinking, and leisure-time exercise), BMI, and total calorie intake | FDR<0.1 |
| Zhang et al. 2018^31^ |  | Sampled and stored at −80 °C immediately | PowerLyzer PowerSoil DNA Isolation Kit (MO BIO, Carlsbad, CA, USA) | Illumina HiSeq 2000; 100bp PE | Around 60 million PE reads | High quality reads were mapped to the recently published reference gut integrated gene catalog (IGC) | In-house pipeline | KEGG | Enterotypes, gender, BMI, age, immune indices and diet history | FDR<0.05 |
| Zhernakova et al. 2016^32^ | Collected and stored in freezer | Within few days transport on dry ice and stored at −80 °C | Described elsewhere | Illumina HiSeq 2000; 50bp PE | An average of 3.0 Gb of data (about 32.3 million reads) per sample | Bowtie2 (version 2.1.0) | MetaPhlan 2.0 | HUMAnN2 | Age, gender, sequencing depth | FDR<0.1 |
| Zou et al. 2020^33^ | Self-collected and then transferred laboratory on dry ice | at −80 °C | Described elsewhere | BGISEQ-500, 100bp PE | Raw reads: average 85117024,89 reads per sample; high quality reads: 83651642,63 reads per sample | SOAP2.22 | SOAP2.22 | KEGG | BMI loss ratio | FDR<0.05 |

*Abbreviations*: BMI: body mass index; bp: base-pair; FDR: false discovery rate; h: hours; KEGG: Kyoto Encyclopedia of Genes and Genomes; PE: Paired-end; SFA: saturated fatty acids; SE: Single-end

**References**

1. Ang QY, Alexander M, Newman JC, et al. Ketogenic Diets Alter the Gut Microbiome Resulting in Decreased Intestinal Th17 Cells. *Cell*. 2020;181(6):1263-1275.e16. doi:10.1016/j.cell.2020.04.027

2. Asnicar F, Berry SE, Valdes AM, et al. Microbiome connections with host metabolism and habitual diet from 1,098 deeply phenotyped individuals. *Nat Med*. 2021;27(2):321-332. doi:10.1038/s41591-020-01183-8

3. Barber C, Mego M, Sabater C, et al. Differential effects of western and mediterranean-type diets on gut microbiota: A metagenomics and metabolomics approach. *Nutrients*. 2021;13(8). doi:10.3390/nu13082638

4. Basolo A, Hohenadel M, Ang QY, et al. Effects of underfeeding and oral vancomycin on gut microbiome and nutrient absorption in humans. *Nat Med*. 2020;26(4):589-598. doi:10.1038/s41591-020-0801-z

5. Benítez-Páez A, Hess AL, Krautbauer S, et al. Sex, Food, and the Gut Microbiota: Disparate Response to Caloric Restriction Diet with Fiber Supplementation in Women and Men. *Mol Nutr Food Res*. 2021;65(8). doi:10.1002/mnfr.202000996

6. Bolte LA, Vich Vila A, Imhann F, et al. Long-term dietary patterns are associated with pro-inflammatory and anti-inflammatory features of the gut microbiome. *Gut*. 2021;70(7):1287-1298. doi:10.1136/gutjnl-2020-322670

7. Cotillard A, Kennedy SP, Kong LC, et al. Dietary intervention impact on gut microbial gene richness. *Nature*. 2013;500(7464):585-588. doi:10.1038/nature12480

8. De Angelis M, Ferrocino I, Calabrese FM, et al. Diet influences the functions of the human intestinal microbiome. *Sci Rep*. 2020;10(1). doi:10.1038/s41598-020-61192-y

9. Hansen LBS, Roager HM, Søndertoft NB, et al. A low-gluten diet induces changes in the intestinal microbiome of healthy Danish adults. *Nat Commun*. 2018;9(1). doi:10.1038/s41467-018-07019-x

10. Kong LC, Holmes BA, Cotillard A, et al. Dietary patterns differently associate with inflammation and gut microbiota in overweight and obese subjects. *PLoS One*. 2014;9(10). doi:10.1371/journal.pone.0109434

11. Kushugulova A, Forslund SK, Costea PI, et al. Metagenomic analysis of gut microbial communities from a Central Asian population. *BMJ Open*. 2018;8(7). doi:10.1136/bmjopen-2018-021682

12. Larke JA, Bacalzo N, Castillo JJ, et al. Dietary Intake of Monosaccharides from Foods is Associated with Characteristics of the Gut Microbiota and Gastrointestinal Inflammation in Healthy US Adults. *Journal of Nutrition*. 2023;153(1):106-119. doi:10.1016/j.tjnut.2022.12.008

13. Le Roy CI, Kurilshikov A, Leeming ER, et al. Yoghurt consumption is associated with changes in the composition of the human gut microbiome and metabolome. *BMC Microbiol*. 2022;22(1). doi:10.1186/s12866-021-02364-2

14. Li J, Hou Q, Zhang J, et al. Carbohydrate staple food modulates gut microbiota of Mongolians in China. *Front Microbiol*. 2017;8(MAR). doi:10.3389/fmicb.2017.00484

15. Li Y, Wang DD, Satija A, et al. Plant-Based Diet Index and Metabolic Risk in Men: Exploring the Role of      the Gut Microbiome. *J Nutr*. 2021;151(9):2780-2789. doi:10.1093/jn/nxab175

16. Liu W, Zhang J, Wu C, et al. Unique Features of Ethnic Mongolian Gut Microbiome revealed by metagenomic analysis. *Sci Rep*. 2016;6. doi:10.1038/srep34826

17. Ma W, Nguyen LH, Song M, et al. Dietary fiber intake, the gut microbiome, and chronic systemic inflammation in a cohort of adult men. *Genome Med*. 2021;13(1). doi:10.1186/s13073-021-00921-y

18. Meslier V, Laiola M, Roager HM, et al. Mediterranean diet intervention in overweight and obese subjects lowers plasma cholesterol and causes changes in the gut microbiome and metabolome independently of energy intake. *Gut*. 2020;69(7):1258-1268. doi:10.1136/gutjnl-2019-320438

19. Oliver A, Chase AB, Weihe C, et al. High-Fiber, Whole-Food Dietary Intervention Alters the Human Gut Microbiome but Not Fecal Short-Chain Fatty Acids. *mSystems*. 2021;6(2). doi:10.1128/msystems.00115-21

20. Rehner J, Schmartz GP, Kramer T, Keller V, Keller A, Becker SL. The Effect of a Planetary Health Diet on the Human Gut Microbiome: A Descriptive Analysis. *Nutrients*. 2023;15(8). doi:10.3390/nu15081924

21. Rinott E, Youngster I, Yaskolka Meir A, et al. Effects of Diet-Modulated Autologous Fecal Microbiota Transplantation on Weight Regain. *Gastroenterology*. 2021;160(1):158-173.e10. doi:10.1053/j.gastro.2020.08.041

22. Munch Roager H, Vogt JK, Kristensen M, et al. Whole grain-rich diet reduces body weight and systemic low-grade inflammation without inducing major changes of the gut microbiome: A randomised cross-over trial. *Gut*. 2019;68(1):83-93. doi:10.1136/gutjnl-2017-314786

23. Shetty SA, Stege PB, Hordijk J, et al. Species-Specific Patterns of Gut Metabolic Modules in Dutch Individuals with Different Dietary Habits. *mSphere*. 2022;7(6). doi:10.1128/msphere.00512-22

24. Stege PB, Hordijk J, Shetty SA, et al. Impact of long-term dietary habits on the human gut resistome in the Dutch population. *Sci Rep*. 2022;12(1). doi:10.1038/s41598-022-05817-4

25. Tarallo S, Ferrero G, De Filippis F, et al. Stool microRNA profiles reflect different dietary and gut microbiome patterns in healthy individuals. *Gut*. 2022;71(7):1302-1314. doi:10.1136/gutjnl-2021-325168

26. Taylor BC, Lejzerowicz F, Poirel M, et al. Consumption of Fermented Foods Is Associated with Systematic Differences in the Gut Microbiome and Metabolome. *mSystems*. 2020;5(2). doi:10.1128/msystems.00901-19

27. Wang F, Wan Y, Yin K, et al. Lower Circulating Branched-Chain Amino Acid Concentrations Among Vegetarians are Associated with Changes in Gut Microbial Composition and Function. *Mol Nutr Food Res*. 2019;63(24):e1900612. doi:10.1002/mnfr.201900612

28. Wu GD, Chen J, Hoffmann C, et al. Linking long-term dietary patterns with gut microbial enterotypes. *Science (1979)*. 2011;334(6052):105-108. doi:10.1126/science.1208344

29. Xiao C, Wang JT, Su C, et al. Associations of dietary diversity with the gut microbiome, fecal metabolites, and host metabolism: results from 2 prospective Chinese cohorts. *American Journal of Clinical Nutrition*. 2022;116(4):1049-1058. doi:10.1093/ajcn/nqac178

30. Yu D, Yang Y, Long J, et al. Long-term Diet Quality and Gut Microbiome Functionality: A Prospective, Shotgun Metagenomic Study among Urban Chinese Adults. *Curr Dev Nutr*. 2021;5(4). doi:10.1093/cdn/nzab026

31. Zhang C, Björkman A, Cai K, et al. Impact of a 3-months vegetarian diet on the gut microbiota and immune repertoire. *Front Immunol*. 2018;9(APR). doi:10.3389/fimmu.2018.00908

32. Zhernakova A, Kurilshikov A, Bonder MJ, et al. Population-based metagenomics analysis reveals markers for gut microbiome composition and diversity. *Science (1979)*. 2016;352(6285):565-569. doi:10.1126/science.aad3369

33. Zou H, Wang D, Ren H, et al. Effect of caloric restriction on BMI, gut microbiota, and blood amino acid levels in non-obese adults. *Nutrients*. 2020;12(3). doi:10.3390/nu12030631
